# Trends in Stroke Thrombolysis Care Metrics and Outcomes by Race and Ethnicity, 2003-2021

**DOI:** 10.1001/jamanetworkopen.2023.52927

**Published:** 2024-02-07

**Authors:** Shumei Man, Nicole Solomon, Brian Mac Grory, Brooke Alhanti, Jeffrey L. Saver, Eric E. Smith, Ying Xian, Deepak L. Bhatt, Lee H. Schwamm, Ken Uchino, Gregg C. Fonarow

**Affiliations:** 1 Cerebrovascular Center, Department of Neurology, Neurological Institute, Cleveland Clinic, Cleveland, Ohio; 2 Duke Clinical Research Institute, Duke University, Durham, North Carolina; 3 Department of Neurology, Duke University School of Medicine, Durham, North Carolina; 4 Department of Neurology, University of California, Los Angeles; 5 Hotchkiss Brain Institute, Department of Clinical Neurosciences, University of Calgary, Calgary, Alberta, Canada; 6 Department of Neurology, University of Texas Southwestern Medical Center, Dallas, Texas; 7 Mount Sinai Fuster Heart Hospital, Icahn School of Medicine at Mount Sinai, New York, New York; 8 Department of Neurology, Massachusetts General Hospital, Boston, Massachusetts; 9 Division of Cardiology, University of California, Los Angeles

## Abstract

**Importance:**

Understanding is needed of racial and ethnic–specific trends in care quality and outcomes associated with the US nationwide quality initiative Target: Stroke (TS) in targeting thrombolysis treatment for acute ischemic stroke.

**Objective:**

To examine whether the TS quality initiative was associated with improvement in thrombolysis metrics and outcomes across racial and ethnic groups.

**Design, Setting, and Participants:**

This retrospective cohort study included patients who presented within 4.5 hours of ischemic stroke onset at hospitals participating in the Get With The Guidelines–Stroke initiative from January 1, 2003, to December 31, 2021. The data analysis was performed between December 15, 2022, and November 27, 2023.

**Exposures:**

TS phases I (2010-2013), II (2014-2018), and III (2019-2021).

**Main Outcomes and Measures:**

The primary outcomes were thrombolysis rates and time metrics. Patient function and mortality were secondary outcomes.

**Results:**

Analyses included 1 189 234 patients, of whom 1 053 539 arrived to the hospital within 4.5 hours. The cohort included 50.4% female and 49.6% male patients and 2.8% Asian [median (IQR) age, 72 (61-82) years], 15.2% Black [median (IQR) age, 64 (54-75) years], 7.3% Hispanic [median (IQR) age, 68 (56-79) years], and 74.1% White [median (IQR) age, 75 (63-84) years] patients). Unadjusted thrombolysis rates increased in both the pre-TS (2003-2009) and TS periods in all racial and ethnic groups from 10% to 15% in 2003 to 43% to 46% in 2021, but disparities were observed in adjusted analyses and persisted in TS phase III, with Asian, Black, and Hispanic patients having significantly lower odds of receiving thrombolysis than White patients (adjusted odds ratio, 0.85 [95% CI, 0.81-0.90], 0.76 [95% CI, 0.74-0.78], and 0.86 [95% CI, 0.83-0.89], respectively). Door-to-needle (DTN) times improved in all racial and ethnic groups during TS, with DTN times of 60 minutes or less increasing from 26% to 28% in 2009 to 66% to 72% in 2021. However, in adjusted analyses, racial and ethnic disparities emerged. During TS phase III, compared with White patients, Asian, Black, and Hispanic patients had significantly lower odds of receiving thrombolysis with a DTN time of 60 minutes or less compared with White patients (risk-adjusted odds ratios, 0.91 [95% CI, 0.84-0.98], 0.78 [95% CI, 0.75-0.81], and 0.87 [95% CI, 0.83-0.92], respectively). During TS, clinical outcomes improved for all racial and ethnic groups from pre-TS, with TS phase III showing higher odds of ambulation at discharge among Asian, Black, Hispanic, and White patients. Asian, Black, and Hispanic patients were less likely to present within 4.5 hours.

**Conclusions and Relevance:**

In this cohort study of patients with ischemic stroke, the TS quality initiative was associated with improvement in thrombolysis frequency, timeliness, and outcomes for all racial and ethnic groups. However, disparities persisted, indicating a need for further interventions.

## Key Points

**Question:**

Did thrombolysis metrics and outcomes improve for all patients following implementation of the Target: Stroke quality initiative, or were there disparities across racial and ethnic groups?

**Findings:**

This cohort study of more than 1 million patients with stroke showed substantial improvements in thrombolysis frequency, timeliness, and outcomes across all races and ethnicities during the 2010-2021 Target: Stroke period. Asian, Black, and Hispanic patients had lower odds of arriving within 4.5 hours and receiving thrombolysis, with longer time to treatment among those treated.

**Meaning:**

These findings suggest that Target: Stroke was associated with large improvements in thrombolytic treatment for all racial and ethnic groups, yet residual disparities persist, indicating a need for further health equity interventions.

## Teaser

This quality improvement study investigates the thrombolysis metrics and outcomes associated with the Target: Stroke initiative by race and ethnicity in a national cohort of US hospitals.

## Introduction

Intravenous thrombolysis (IVT) has been shown to improve outcomes in appropriately selected patients when administered within 4.5 hours of stroke onset.^1,2^ The benefits of thrombolysis are time dependent, and earlier treatment is associated with better outcomes.^3,4,5,6,7^ However, historically, only 20% to 30% of patients received thrombolysis within a door-to-needle (DTN) time of 60 minutes.^8^ To assist hospitals in delivering timely thrombolytic treatment and avoiding delays, the American Heart Association and American Stroke Association launched a nationwide quality initiative, Target: Stroke (TS), in January 2010.^8^ Target: Stroke provides hospitals with best practice strategies, goals, and recognitions to accelerate thrombolytic administration.^8,9^

The treatment time goals of TS were iteratively made more stringent, as follows: phase I (TS:I) (2010-2013), DTN within 60 minutes in at least 50% of patients; phase II (TS:II) (2014-2018), DTN within 60 minutes in at least 75% and within 45 minutes in at least 50% of patients; and phase III (TS:III) (2019 onward), DTN within 60, 45, and 30 minutes in at least 85%, 75%, and 50% of patients, respectively. The initial launch of TS:I was associated with a nearly 2-fold increase in DTN of 60 minutes or less, lower in-hospital mortality, and increased discharges home.^8,10,11^ The TS:II was associated with further improvement in thrombolytic treatment speed, leading to the launch of TS:III with even more stringent treatment time goals.^12,13,14^ Changes in thrombolysis care and outcomes during TS:III have not been examined.

In addition, there have been reports of racial and ethnic disparities of thrombolysis use in stroke in the past decade.^15,16,17,18,19,20,21,22^ A study of National Inpatient Sample 2004-2010 data showed that among patients hospitalized for ischemic stroke, without accounting for presentation timeliness, Black and Hispanic patients were less likely to be treated with IVT than White patients.^19^ Another study using later 2009-2018 National Inpatient Sample data showed improvements but that Black individuals continued to receive less thrombolytic treatment than White individuals.^20^ The administrative data studies had limitations, including an inability to examine the contributions of time of presentation to observed rates of treatment. Of particular interest is whether presentation within or beyond the 4.5-hour thrombolytic time window contributed to disparities as time of presentation is potentially modifiable by individual and public health education and is known to show variation by race and ethnicity.^23^ Accordingly, the objectives of this study were to evaluate whether TS is associated with equitable improvement in thrombolysis care metrics and outcomes in patients of various races and ethnicities and the presence and sources of any residual disparities.

## Methods

### Data Source and Study Population

This retrospective, observational cohort study examined patients treated for acute ischemic stroke at hospitals participating in Get With The Guidelines–Stroke (GWTG-Stroke) from January 1, 2003, to December 31, 2021. The GWTG-Stroke is a prospective data collection initiative launched by the American Heart Association and American Stroke Association.^24,25^ Patient-level clinical data are collected by trained hospital personnel for consecutive patients treated for acute ischemic stroke.^24,25^ Race and ethnicity data are recorded using the GWTG-Stroke data entry tool, which supports a multiple selection process that includes single race, multiple races, and ethnic categories as well as a separate data element for Hispanic ethnicity (yes vs no or not documented), and collected through various sources, including patient self-designation, administrative personnel during the registration process, and nursing intake forms.^21,26,27^ Each participating hospital received either human research approval to enroll patients without informed consent under the Common Rule or through exemption from their institutional review board. The Duke Clinical Research Institute serves as the data analysis center. Given that the primary purpose of the registry is for quality improvement, a waiver for patient informed consent is granted under the Common Rule. The institutional review board at Duke University Health approved this study. This study follows the Strengthening the Reporting of Observational Studies in Epidemiology (STROBE) reporting guideline.

Detailed information about the GWTG-Stroke database, Target: Stroke, the statistical analysis, and missing data are provided in the eMethods and eFigure 1 in Supplement 1. Patients with in-hospital stroke were excluded, as were patients with unknown or missing age, sex, race and ethnicity, and last known well (LKW) or arrival times. Race and ethnicity information of patients with missing LKW are provided in eTable 1 in Supplement 1. Patients who were transferred in were excluded due to unknown treatment timeliness at the first hospital. Primary analyses were conducted among those arriving at the hospital within 4.5 hours from LKW. The proportion of patients arriving after 4.5 hours was calculated among those with documented LKW, excluding patients with missing or unknown LKW, such as wake-up strokes.

### Outcomes

This study had 4 primary outcomes: (1) thrombolysis rates among patients arriving within 4.5 hours; (2) proportion of patients receiving thrombolysis with a DTN of 60 minutes or less; (3) among patients arriving by 2 hours without thrombolytic contraindications, thrombolytic treatment by 3 hours; and (4) among patients arriving by 3.5 hours without thrombolytic contraindications, thrombolytic treatment by 4.5 hours. Secondary process metric outcomes were the proportion of patients receiving thrombolysis with DTN time within 30 and 45 minutes. Secondary clinical efficacy outcomes were discharge home rate among all IVT-treated patients and independent ambulation at discharge among IVT-treated patients discharged alive. Secondary clinical safety outcomes among all IVT-treated patients were in-hospital mortality and combined in-hospital mortality and discharge to hospice.

### Covariates

Covariates used in the adjusted models included patient demographics (age, sex, and race and ethnicity [categorized as Asian; Black; Hispanic; Native American, Pacific Islander, or other race and ethnicity; and White]); medical history (atrial fibrillation or flutter, previous stroke or transient ischemic attack, history of coronary artery disease or myocardial infarction, carotid stenosis, diabetes, peripheral vascular disease, hypertension, dyslipidemia, and smoking); admission variables (arrival by emergency medical service [EMS], onset-to-arrival time, arrival during off hours, antiplatelet or anticoagulant prior to admission, and stroke severity as measured by initial National Institutes of Health Stroke Scale [NIHSS]); and hospital characteristics (census region, rural location, total bed number, annual ischemic stroke volume, annual IVT volume, teaching status, and stroke center certification). These variables have been previously used and validated in GWTG-Stroke analyses.^28,29,30,31,32,33^

### Statistical Analysis

Annual thrombolytic treatment rates and outcomes in Asian, Hispanic, non-Hispanic Black, and non-Hispanic White patients are depicted graphically. The number of patients identifying as Native American, Pacific Islander, or other race and ethnicity was low in the study cohort, which did not permit modeling analysis. Differences in key metrics by race and ethnicity, relative to White patients, were estimated using multivariable logistic regression models. We used stepwise adjustment as follows: unadjusted and adjusted for demographics, adding medical history, then admission variables, and then hospital factors. The models accounted for within- and across-hospital variability with hospital-specific random intercepts.

A multivariable logistic regression model with random intercepts was used to compare the outcomes in TS:I (January 1, 2010-December 31, 2013), TS:II (January 1, 2014-December 31, 2018), and TS:III (January 1, 2019-December 31, 2021) vs the pre-TS period (January 1, 2003-December 31, 2009) within each race and ethnicity and the interaction of race and ethnicity with TS. Logistic regression models were also used to estimate the gaps between Asian, Black, and Hispanic patients and White patients during the pre-TS period and TS:III. The models were adjusted for the covariates listed above, and restricted cubic splines were applied to continuous variables as needed.

Patients with missing data for sex, age, race and ethnicity, and thrombolysis timeliness were excluded from the analysis. Missing rates of key patient and hospital characteristics were low (eTables 2 and 3 in Supplement 1). Covariates with 25% or more missingness were excluded in the adjusted analyses. Due to computational constraints, for remaining covariates with missing data, single imputation to the race-specific mode or median was performed. The NIHSS score was not imputed.

All statistical analyses were performed from December 15, 2022, to November 27, 2023, using SAS, version 9.4 software (SAS Institute Inc). Odds ratios (ORs) and 95% CIs from the logistic regression models are reported for the parameter estimates. For the interaction analysis, *P* values were derived from the logistic regression, and 2-sided *P* < .05 was considered statistically significant.

## Results

### Baseline Characteristics

As shown in eFigure 2 in Supplement 1, after applying study entry criteria, the study included 1 189 234 patients with ischemic stroke arriving at GWTG-Stroke–participating hospitals within 4.5 hours from stroke onset. Among these patients, 50.4% were female and 49.6% male; 2.8% were Asian (median [IQR] age, 72 [61-82] years); 15.2% were Black (median [IQR] age, 64 [54-75] years); 7.3% were Hispanic (median [IQR] age, 68 [56-79] years); 0.6% were Native American, Pacific Islander, or other race or ethnicity (median [IQR] age, 66 [55-76] years); and 74.1% were White (median [IQR] age, 75 [63-84] years). Patient and hospital characteristics along with descriptive outcomes by race and ethnicity are provided in Table 1. Eleven percent of Hispanic patients and 8.6% of Black patients were uninsured, compared with 3.3% of White patients. The median (IQR) presenting NIHSS score was 5 (2-12) across races and ethnicities.

**Table 1.** Patient and Hospital Characteristics by Race and Ethnicity With Onset to Arrival of 4.5 Hours or Less

Characteristic	No. (%)					
	Overall	Asian	Black	Hispanic	Native American, Pacific Islander, or other^a^	White
No. of patients	1 189 234 (100)	33 375 (2.8)	180 315 (15.2)	86 831 (7.3)	7052 (0.6)	881 661 (74.1)
**Demographics**						
Age, median (IQR), y	73 (61-83)	72 (61-82)	64 (54-75)	68 (56-79)	66 (55-76)	75 (63-84)
Sex						
Female	598 888 (50.4)	16 001 (47.9)	95 453 (52.9)	42 552 (49.0)	3669 (52.0)	441 213 (50.0)
Male	590 346 (49.6)	17 374 (52.1)	84 862 (47.1)	44 279 (51.0)	3383 (48.0)	440 448 (50.0)
Insurance						
Medicaid	98 883 (10.5)	5097 (19.9)	29 563 (20.5)	14 482 (21.6)	1151 (20.9)	48 590 (7.0)
Medicare	379 625 (40.3)	8238 (32.1)	47 177 (32.7)	21 749 (32.5)	1575 (28.6)	300 886 (43.1)
Private, VA, CHAMPUS, or other	411 984 (43.8)	10 536 (41.1)	53 598 (37.1)	22 778 (34.0)	2390 (43.3)	322 682 (46.2)
Self-pay	44 259 (4.7)	1522 (5.9)	12 353 (8.6)	7335 (11.0)	332 (6.0)	22 717 (3.3)
Unknown	6316 (0.7)	270 (1.1)	1623 (1.1)	620 (0.9)	67 (1.2)	3736 (0.5)
**Comorbidities**						
Atrial fibrillation or flutter	257 463 (21.6)	6964 (20.9)	23 196 (12.9)	13 673 (15.7)	1274 (18.1)	212 356 (24.1)
Prior stroke	279 354 (23.5)	7532 (22.6)	54 440 (30.2)	21 778 (25.1)	1795 (25.5)	193 809 (22.0)
Prior transient ischemic attack	109 934 (9.2)	1728 (5.2)	13 692 (7.6)	5909 (6.8)	540 (7.7)	88 065 (10.0)
Coronary artery disease or prior myocardial infarction	292 375 (24.6)	6020 (18.0)	35 220 (19.5)	17 225 (19.8)	1562 (22.1)	232 348 (26.4)
Carotid stenosis	42 552 (3.6)	574 (1.7)	3034 (1.7)	1799 (2.1)	182 (2.6)	36 963 (4.2)
Heart failure	111 147 (9.3)	2218 (6.6)	22 433 (12.4)	6388 (7.4)	736 (10.4)	79 372 (9.0)
Kidney insufficiency	75 446 (6.3)	2183 (6.5)	15 517 (8.6)	5442 (6.3)	601 (8.5)	51 703 (5.9)
Diabetes	352 867 (29.7)	11 484 (34.4)	69 214 (38.4)	36 271 (41.8)	2947 (41.8)	232 951 (26.4)
Peripheral vascular disease	48 319 (4.1)	568 (1.7)	6260 (3.5)	2503 (2.9)	197 (2.8)	38 791 (4.4)
Hypertension	879 209 (73.9)	25 130 (75.3)	146 131 (81.0)	64 008 (73.7)	5256 (74.5)	638 684 (72.4)
Dyslipidemia	539 602 (45.4)	15 274 (45.8)	71 535 (39.7)	36 272 (41.8)	3160 (44.8)	413 361 (46.9)
Prosthetic heart valve	17 953 (1.5)	344 (1.0)	1535 (0.9)	970 (1.1)	119 (1.7)	14 985 (1.7)
Smoker	184 030 (15.5)	2914 (8.7)	39 569 (21.9)	10 050 (11.6)	1321 (18.7)	130 176 (14.8)
**Census data**						
Region						
Northeast	271 291 (22.8)	6512 (19.5)	35 355 (19.6)	15 836 (18.2)	672 (9.5)	212 916 (24.1)
Midwest	227 868 (19.2)	2584 (7.7)	32 690 (18.1)	5706 (6.6)	844 (12.0)	186 044 (21.1)
South	451 181 (37.9)	5268 (15.8)	96 549 (53.5)	35 876 (41.3)	1488 (21.1)	312 000 (35.4)
West	238 894 (20.1)	19 011 (57.0)	15 721 (8.7)	29 413 (33.9)	4048 (57.4)	170 701 (19.4)
No. of beds, median (IQR)	344 (218-524)	327 (215-507)	398 (255-630)	360 (251-548)	303 (199-508)	331 (207-506)
Rural location	61 925 (5.2)	614 (1.8)	6012 (3.3)	962 (1.1)	712 (10.1)	53 625 (6.1)
Teaching hospital	851 665 (71.6)	24 897 (74.6)	141 880 (78.7)	65 953 (76.0)	5081 (72.1)	613 854 (69.6)
Annual ischemic stroke volume, median No. of patients (IQR)	270 (168-429)	276 (183-421)	300 (191-464)	277 (175-433)	256 (162-414)	263 (162-422)
Annual IVT volume	28 (14-51)	33 (18-54)	31 (17-56)	34 (18-59)	29 (15-50)	27 (14-50)
Comprehensive stroke center	259 258 (21.8)	8748 (26.2)	49 697 (27.6)	20 175 (23.2)	1575 (22.3)	179 063 (20.3)
Primary stroke center	663 607 (55.8)	19 160 (57.4)	90 977 (50.5)	48 753 (56.1)	3891 (55.2)	500 826 (56.8)
No stroke center	266 369 (22.4)	5467 (16.4)	39 641 (22.0)	17 903 (20.6)	1586 (22.5)	201 772 (22.9)
**Arrival data**						
Onset to arrival, median (IQR), min	76 (46-136)	77 (45-143)	76 (45-141)	74 (44-137)	76 (45-139)	76 (47-135)
≤60	467 334 (39.3)	13 137 (39.4)	71 701 (39.8)	36 051 (41.5)	2809 (39.8)	343 636 (39.0)
61-120	367 141 (30.9)	9619 (28.8)	53 035 (29.4)	25 032 (28.8)	2095 (29.7)	277 360 (31.5)
121-210	246 460 (20.7)	7253 (21.7)	37 634 (20.9)	17 687 (20.4)	1491 (21.1)	182 395 (20.7)
>210	108 299 (9.1)	3366 (10.1)	17 945 (10.0)	8061 (9.3)	657 (9.3)	78 270 (8.9)
NIHSS, No. of patients	1 059 773	30 086	161 936	79 266	6234	782 251
Median score (IQR)	5 (2-12)	6 (2-14)	6 (2-12)	5 (2-13)	5 (2-13)	5 (2-12)
Off-hour arrival^b^	648 118 (54.5)	19 038 (57.0)	101 143 (56.1)	48 220 (55.5)	3953 (56.1)	475 764 (54.0)
Arrival mode						
EMS	830 714 (69.9)	22 788 (68.3)	127 814 (70.9)	57 272 (66.0)	4651 (66.0)	618 189 (70.1)
Private vehicle, taxi, other	320 368 (26.9)	9474 (28.4)	46 994 (26.1)	27 036 (31.1)	2085 (29.6)	234 779 (26.6)
Mobile stroke unit	1722 (0.1)	53 (0.2)	404 (0.2)	135 (0.2)	7 (0.1)	1123 (0.1)
**Thrombolysis metrics**						
Treatment rate	429 370 (36.1)	13 007 (39.0)	68 114 (37.8)	34 866 (40.2)	2727 (38.7)	310 656 (35.2)
Door to needle, min	55 (40-78)	53 (38-74)	57 (41-80)	54 (39-76)	55 (39-77)	56 (40-77)
≤30	51 407 (12.0)	1813 (13.9)	7735 (11.4)	4760 (13.7)	381 (14.0)	36 718 (11.8)
31-45	95 437 (22.2)	3121 (24.0)	14 694 (21.6)	7762 (22.3)	600 (22.0)	69 260 (22.3)
46-60	102 395 (23.8)	3156 (24.3)	15 999 (23.5)	8437 (24.2)	623 (22.8)	74 180 (23.9)
>60	180 131 (42.0)	4917 (37.8)	29 686 (43.6)	13 907 (39.9)	1123 (41.2)	130 498 (42.0)
Onset to needle, min	130 (96-172)	127 (93-173)	132 (97-175)	127 (93-171)	131 (95-175)	130 (97-171)
**Documented reason for not treated with IVT**						
Relative exclusions	219 969 (18.5)	6370 (19.1)	33 332 (18.5)	16 194 (18.7)	1330 (18.9)	162 743 (18.5)
Patient or family refusal	48 216 (4.1)	1183 (3.5)	6982 (3.9)	3002 (3.5)	245 (3.5)	36 804 (4.2)
**Clinical efficacy and safety outcomes among patients treated with thrombolysis**						
Discharge destination						
Home	577 441 (50.8)	16 764 (53.2)	47 329 (57.0)	90 118 (51.8)	3745 (55.7)	419 485 (49.8)
Postacute facility^c^	383 654 (33.7)	10 335 (32.8)	24 416 (29.4)	61 789 (35.5)	1941 (28.9)	285 173 (33.9)
Hospice	53 541 (4.7)	1243 (3.9)	3199 (3.9)	4170 (2.4)	204 (3.0)	44 725 (5.3)
Acute facility	105 003 (9.2)	2885 (9.1)	6734 (8.1)	14 435 (8.3)	717 (10.7)	80 232 (9.5)
Ambulatory status at discharge						
Independent	521 256 (43.8)	14 411 (43.2)	38 707 (44.6)	80 606 (44.7)	3206 (45.5)	384 326 (43.6)
With assistance	270 657 (22.8)	7680 (23.0)	19 731 (22.7)	42 212 (23.4)	1469 (20.8)	199 565 (22.6)
Not ambulatory	144 256 (12.1)	4461 (13.4)	10 930 (12.6)	22 633 (12.6)	732 (10.4)	105 500 (12.0)
Symptomatic intracerebral hemorrhage	14 832 (3.5)	542 (4.2)	1225 (3.5)	2260 (3.3)	76 (2.8)	10 729 (3.5)
Thrombolytic complication	27 703 (6.5)	955 (7.3)	2279 (6.5)	4454 (6.5)	179 (6.6)	19 836 (6.4)
In-hospital death	51 673 (4.3)	1836 (5.5)	3846 (4.4)	6277 (3.5)	328 (4.7)	39 386 (4.5)
Death or hospice^d^	105 214 (8.8)	3079 (9.2)	7045 (8.1)	10 447 (5.8)	532 (7.5)	84 111 (9.5)

Abbreviations: CHAMPUS, Civilian Health and Medical Program of the Uniformed Services; EMS, emergency medical service; IVT, intravenous thrombolysis; NIHSS, National Institutes of Health Stroke Scale; VA, Veterans Affairs.

^a^ Other race and ethnicity included Native Hawaiian and those for whom the category “specify other race” was selected on the Get With The Guideline–Stroke case report form.

^b^ Regular working hours were defined as 7:00 AM to 6:00 PM, Monday through Friday on nonholidays. Arriving outside of these hours were considered off hours.

^c^ Included inpatient rehabilitation facility, skilled nursing facility, intermediate care facility, and long-term-care hospital.

^d^ Included home hospice and facility hospice.

### Disparities in Arrival Times and Postarrival Thrombolysis

**Figure 1.**
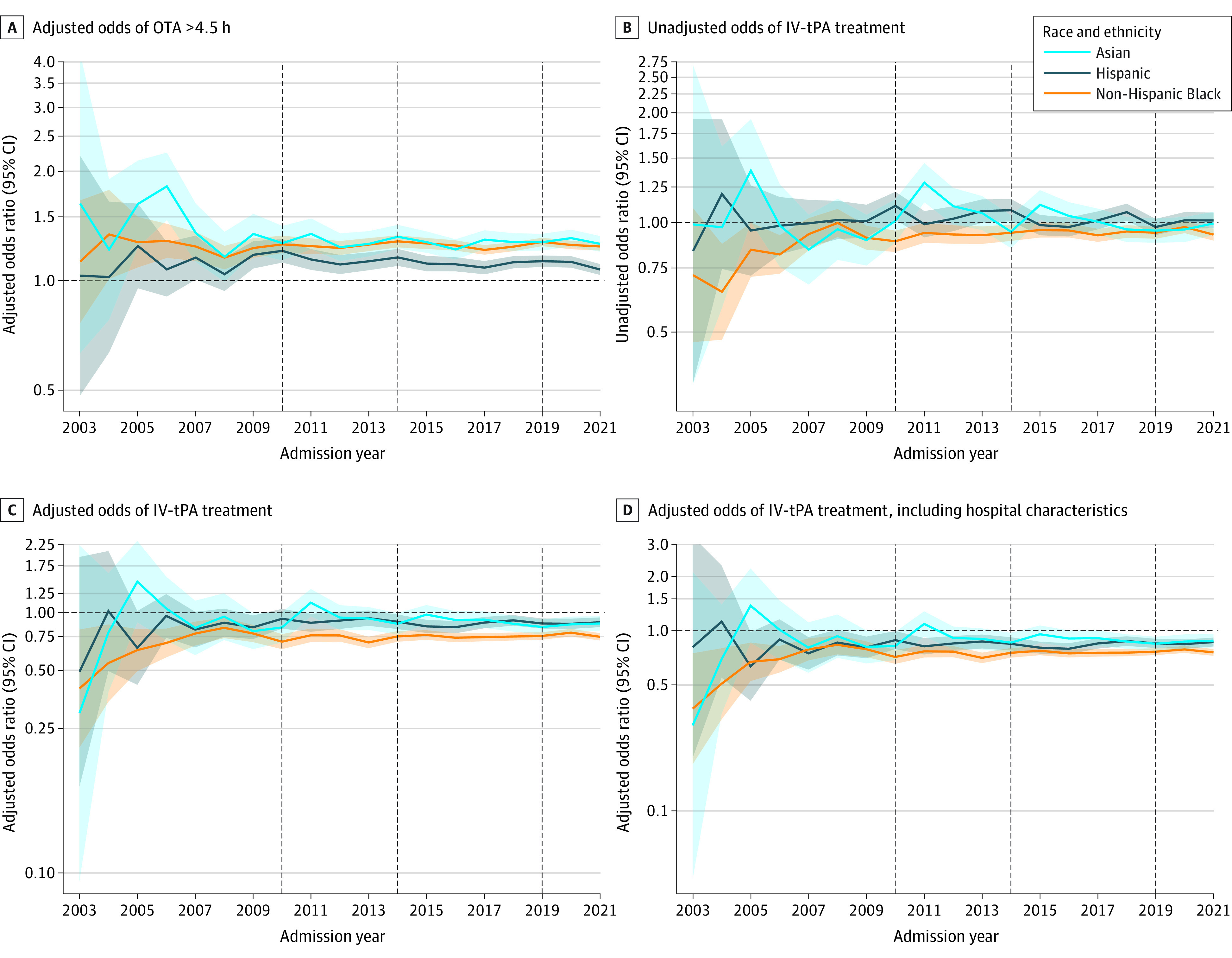
Racial and Ethnic Disparities in Arrival Time and Thrombolysis Rates Among Patients Arriving Within 4.5 Hours With Stepwise Adjustment The reference group is non-Hispanic White patients. Panel A excludes those with unknown or missing last known well times. Panel C is adjusted for demographics, medical history, and admission variables. Panel D is adjusted for demographics, medical history, admission variables, and hospital characteristics. Shaded areas represent 95% CIs, and the vertical dashed lines represent the beginning of each Target: Stroke phase, with phase 1 in January 2010, phase 2 in January 2014, and phase 3 in January 2019. IV-tPA indicates intravenous tissue plasminogen activator; OTA, onset to arrival.

Throughout the study period, Asian, Black, and Hispanic patients were more likely to arrive at the hospital after 4.5 hours than White patients in both unadjusted (eFigure 3A and B in Supplement 1) and adjusted analyses (Figure 1A). Delayed arrival increased for all races and ethnicities from 2004 to 2021, parallel to an increasing number of small and rural hospitals joining GWTG-Stroke (eFigure 4A-C in Supplement 1). It is possible that more patients with long travel times were treated at GWTG-Stroke hospitals. Among the 1 053 539 patients presenting within 4.5 hours, unadjusted thrombolysis rates increased from 10% to 15% in 2003 to 43% to 46% in 2021 across all racial and ethnic groups, without significant disparities observed (eFigure 5 in Supplement 1; Figure 1B). However, disparities emerged after adjusting for patient demographics, comorbidities, and admission variables (Figure 1C) and hospital characteristics (Figure 1D), with Asian, Black, and Hispanic patients having lower odds of receiving thrombolysis throughout the period of 2007-2021 compared with White patients (adjusted ORs [AORs], 0.85 [95% CI, 0.81-0.90] for Asian, 0.76 [95% CI, 0.74-0.78] for Black, and 0.86 [95% CI, 0.83-0.89] for Hispanic).

### Improvements in Thrombolysis Frequency, Timeliness, and Outcomes for All Races and Ethnicities

**Figure 2.**
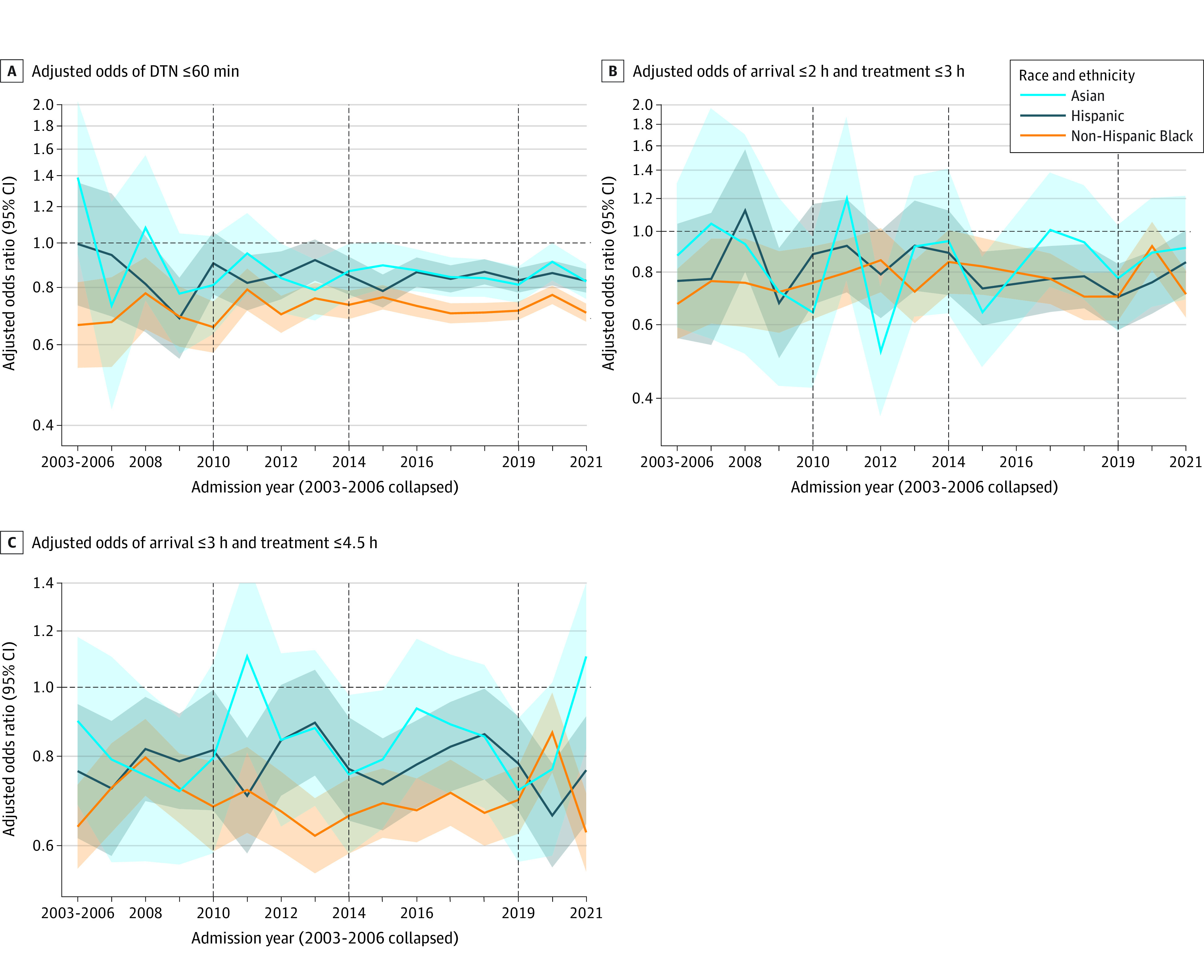
Racial and Ethnic Disparities in Thrombolysis Arrival-to-Treatment Time Metrics The reference group is non-Hispanic White patients. Shaded areas represent 95% CIs, and the vertical dashed lines represent the beginning of each Target: Stroke phase, with phase 1 in January 2010, phase 2 in January 2014, and phase 3 in January 2019. DTN indicates door to needle.

Among the 357 633 patients receiving thrombolysis within 4.5 hours, DTN times were relatively unchanged throughout the 2003-2009 pre-TS period and improved substantially in all racial and ethnic groups during the TS period (eFigure 6A in Supplement 1). The proportion with DTN time of 60 minutes or less sharply increased throughout TS:I, followed by a slower but continuous increase throughout TS:II and TS:III, with an overall change from 26% to 28% in 2009 to 66% to 72% in 2021. The DTN time of 45 minutes or less showed a similar pattern of improvement. The DTN time of 30 minutes had a small increase in TS:I but substantial increases in TS:II and TS:III, with an overall increase from 3% in 2003 to 18% to 24% in 2021. Substantial improvements in treatment frequency and speed metrics were seen in all racial and ethnic groups, with significant associations between race and ethnicity and TS that favored certain racial and ethnic populations compared with the White population in each phase (Table 2); however disparities were noted (Figure 2; Table 3). After adjusting for patient and hospital factors, compared with pre-TS, TS:III was associated with an increase in thrombolytic treatment rates among all races and ethnicities (AORs, 1.92 [95% CI, 1.66-2.22] for Asian, 1.84 [95% CI, 1.73-1.96] for Black, 2.00 [95% CI, 1.82-2.19] for Hispanic, and 1.68 [95% CI, 1.63-1.72] for White patients). For the speed of thrombolytic treatment, TS:III was associated with an increased odds of DTN times within 60 minutes (AORs, 5.67 [95% CI, 4.49-7.16] for Asian, 4.94 [95% CI, 4.46-5.46] for Black, 6.00 [95% CI, 5.16-6.97] for Hispanic, and 5.35 [95% CI, 5.11-5.60] for White patients) and DTN within 30 minutes (AORs, 8.14 [95% CI, 4.57-14.50] for Asian, 6.11 [95% CI, 4.75-7.85] for Black, 9.35 [95% CI, 6.48-13.51] for Hispanic, and 6.10 [95% CI, 5.50-6.76] for White patients) (Table 2). After risk adjustment, relative to White patients, Asian, Black, and Hispanic patients had lower odds of receiving thrombolysis with a DTN time within 60 minutes (AORs, 0.91 [95% CI, 0.84-0.98] for Asian, 0.78 [95% CI, 0.75-0.81] for Black, and 0.87 [95% CI, 0.83-0.92] for Hispanic), with similar trends observed for DTN times of 45 and 30 minutes or less (Table 3). Clinical efficacy and safety outcomes, including rates of discharge home, independent ambulation at discharge, in-hospital mortality, and combined in-hospital mortality and discharge to hospice rates, showed a relative plateau for all races and ethnicities in pre-TS years 2007-2009, followed by steady improvement throughout TS:1, TS:II, and TS:III numerically (eFigure 7 and eTable 4 in Supplement 1) and after risk adjustment (Table 2).

**Table 2.** Change of Thrombolysis Metrics and Outcomes Over Time by Race and Ethnicity

Outcome	Target: Stroke phase, AOR (95% CI)^a^			*P* value for interaction
	I (2010-2013)	II (2014-2018)	III (2019-2021)	
**IVT treatment metrics**				
Thrombolytic treatment rate (No. of observations modeled, 1 053 539)				
Asian	1.39 (1.21-1.60)	1.79 (1.56-2.06)	1.92 (1.66-2.22)	.003
Black	1.24 (1.18-1.32)	1.66 (1.56-1.76)	1.84 (1.73-1.96)	
Hispanic	1.31 (1.20-1.43)	1.71 (1.57-1.87)	2.00 (1.82-2.19)	
White	1.20 (1.17-1.23)	1.49 (1.45-1.53)	1.68 (1.63-1.72)	
DTN ≤30 min (No. of observations modeled, 414 849)				
Asian	0.96 (0.52-1.77)	3.75 (2.11-6.66)	8.14 (4.57-14.50)	<.001
Black	1.19 (0.92-1.55)	3.45 (2.69-4.42)	6.11 (4.75-7.85)	
Hispanic	1.36 (0.93-1.99)	4.74 (3.29-6.83)	9.35 (6.48-13.51)	
White	1.03 (0.92-1.15)	3.24 (2.93-3.59)	6.10 (5.50-6.76)	
DTN ≤45 min (No. of observations modeled, 414 849)				
Asian	1.59 (1.14-2.22)	5.17 (3.73-7.16)	8.64 (6.21-12.02)	<.001
Black	1.41 (1.23-1.62)	3.86 (3.38-4.42)	5.79 (5.05-6.65)	
Hispanic	1.62 (1.32-1.98)	4.36 (3.58-5.32)	7.60 (6.21-9.30)	
White	1.35 (1.28-1.44)	3.86 (3.64-4.08)	6.26 (5.90-6.64)	
DTN ≤60 min (No. of observations modeled, 414 849)				
Asian	1.48 (1.18-1.86)	4.01 (3.20-5.02)	5.67 (4.49-7.16)	<.001
Black	1.62 (1.47-1.78)	3.73 (3.39-4.12)	4.94 (4.46-5.46)	
Hispanic	1.71 (1.48-1.98)	4.21 (3.64-4.86)	6.00 (5.16-6.97)	
White	1.52 (1.46-1.58)	3.75 (3.60-3.92)	5.35 (5.11-5.60)	
Arrival by 2 h/treat by 3 h (No. of observations modeled, 344 406)				
Asian	1.64 (1.21-2.23)	2.51 (1.82-3.45)	2.89 (2.06-4.07)	.09
Black	1.78 (1.58-2.00)	2.60 (2.30-2.94)	3.08 (2.69-3.53)	
Hispanic	1.89 (1.58-2.26)	2.26 (1.88-2.72)	2.69 (2.21-3.28)	
White	1.74 (1.64-1.83)	2.23 (2.10-2.37)	2.79 (2.60-2.98)	
Arrival by 3.5 h/treat by 4.5 h (No. of observations modeled, 503 693)				
Asian	5.97 (4.90-7.26)	10.33 (8.38-12.74)	25.19 (19.64-32.29)	.07
Black	3.96 (3.66-4.27)	6.70 (6.14-7.30)	15.31 (13.83-16.96)	
Hispanic	4.07 (3.62-4.57)	6.56 (5.77-7.46)	15.41 (13.30-17.86)	
White	4.33 (4.18-4.49)	7.08 (6.79-7.38)	17.45 (16.59-18.36)	
**Clinical efficacy and safety outcomes among patients treated with thrombolysis**				
Discharge home vs facility, hospice, or death (No. of observations modeled, 347 870)^b^				
Asian	1.39 (1.10-1.75)	1.57 (1.24-1.97)	2.07 (1.63-2.63)	.64
Black	1.18 (1.08-1.30)	1.36 (1.24-1.49)	1.64 (1.49-1.81)	
Hispanic	1.31 (1.14-1.51)	1.57 (1.36-1.80)	1.91 (1.65-2.21)	
White	1.16 (1.11-1.21)	1.33 (1.27-1.39)	1.64 (1.56-1.72)	
Independent ambulation at discharge (No. of observations modeled, 313 262)^c^				
Asian	1.48 (1.15-1.90)	1.72 (1.34-2.20)	1.86 (1.44-2.40)	.16
Black	1.06 (0.95-1.17)	1.42 (1.28-1.57)	1.48 (1.33-1.65)	
Hispanic	1.24 (1.06-1.45)	1.47 (1.26-1.72)	1.55 (1.32-1.82)	
White	1.01 (0.97-1.06)	1.34 (1.27-1.40)	1.42 (1.34-1.49)	
In-hospital mortality (No. of observations modeled, 347 870)				
Asian	1.00 (0.71-1.42)	0.81 (0.57-1.14)	0.72 (0.50-1.04)	.87
Black	0.76 (0.65-0.90)	0.65 (0.55-0.77)	0.61 (0.51-0.73)	
Hispanic	0.83 (0.66-1.05)	0.74 (0.58-0.93)	0.68 (0.53-0.88)	
White	0.89 (0.83-0.95)	0.76 (0.71-0.82)	0.69 (0.63-0.74)	
In-hospital mortality or hospice discharge (No. of observations modeled, 347 870)				
Asian	1.01 (0.74-1.39)	0.91 (0.66-1.24)	0.86 (0.62-1.20)	.76
Black	0.83 (0.71-0.97)	0.80 (0.69-0.93)	0.82 (0.70-0.97)	
Hispanic	0.86 (0.70-1.06)	0.85 (0.69-1.04)	0.85 (0.68-1.06)	
White	1.02 (0.97-1.08)	0.95 (0.89-1.01)	0.96 (0.90-1.02)	

Abbreviations: AOR, adjusted odds ratio; DTN, door to needle; IVT, intravenous thrombolysis.

^a^ Reference is before Target: Stroke (2003-2009) for each race and ethnicity.

^b^ Included inpatient rehabilitation facility, skilled nursing facility, intermediate care facility, and long-term-care hospital. Hospice included home hospice and facility hospice.

^c^ Modeled among patients who were discharged alive with discharge ambulatory status documented.

**Table 3.** Thrombolysis Metrics and Outcomes Before Target: Stroke and During Target: Stroke Phase III

Outcome	Target: Stroke phase, AOR (95% CI)^a^	
	Before (2003-2009)	III (2019-2021)
**IVT treatment metrics**		
IVT treatment rate		
Asian	0.88 (0.77-1.00)	0.85 (0.81-0.90)
Black	0.74 (0.70-0.79)	0.76 (0.74-0.78)
Hispanic	0.83 (0.76-0.90)	0.86 (0.83-0.89)
DTN ≤30 min		
Asian	1.06 (0.60-1.88)	0.89 (0.82-0.98)
Black	0.82 (0.63-1.08)	0.77 (0.73-0.80)
Hispanic	0.91 (0.63-1.33)	0.85 (0.80-0.90)
DTN ≤45 min		
Asian	0.85 (0.62-1.16)	0.91 (0.85-0.98)
Black	0.86 (0.74-1.00)	0.75 (0.73-0.78)
Hispanic	0.89 (0.73-1.09)	0.86 (0.82-0.91)
DTN ≤60 min		
Asian	1.08 (0.87-1.33)	0.91 (0.84-0.98)
Black	0.78 (0.71-0.87)	0.78 (0.75-0.81)
Hispanic	0.89 (0.77-1.02)	0.87 (0.83-0.92)
Arrival by 2 h/treat by 3 h		
Asian	0.82 (0.64-1.06)	0.83 (0.70-0.99)
Black	0.72 (0.64-0.80)	0.78 (0.72-0.85)
Hispanic	0.88 (0.75-1.04)	0.76 (0.69-0.85)
Arrival by 3.5 h/treat by 4.5 h		
Asian	0.78 (0.68-0.89)	0.80 (0.69-0.93)
Black	0.71 (0.66-0.76)	0.72 (0.67-0.77)
Hispanic	0.79 (0.72-0.87)	0.75 (0.68-0.83)
**Clinical efficacy and safety outcomes among patients treated with thrombolysis**		
Discharge home vs facility, hospice, or death^b^		
Asian	0.94 (0.75-1.17)	1.12 (1.04-1.22)
Black	0.92 (0.83-1.01)	0.89 (0.86-0.93)
Hispanic	1.13 (0.99-1.30)	1.20 (1.13-1.26)
Independent ambulation at discharge^c^		
Asian	0.71 (0.56-0.89)	0.92 (0.85-1.01)
Black	0.89 (0.81-0.99)	0.83 (0.79-0.87)
Hispanic	0.88 (0.76-1.01)	0.95 (0.89-1.00)
In-hospital mortality		
Asian	0.98 (0.73-1.33)	0.98 (0.84-1.14)
Black	0.93 (0.80-1.08)	0.89 (0.81-0.98)
Hispanic	0.95 (0.77-1.18)	0.95 (0.85-1.06)
In-hospital mortality or hospice discharge		
Asian	0.84 (0.63-1.11)	0.78 (0.69-0.89)
Black	0.75 (0.65-0.87)	0.72 (0.67-0.77)
Hispanic	0.87 (0.72-1.04)	0.89 (0.82-0.97)

Abbreviations: AOR, adjusted odds ratio; DTN, door to needle; IVT, intravenous thrombolysis.

^a^ Reference is White race.

^b^ Included inpatient rehabilitation facility, skilled nursing facility, intermediate care facility, and long-term-care hospital. Hospice included home hospice and facility hospice.

^c^ Modeled among patients who were discharged alive with discharge ambulatory status documented.

### Racial and Ethnic Disparities in Thrombolysis Metrics and Outcomes Before and After TS

Table 3 shows thrombolysis metrics and outcomes in the pre-TS and TS:III periods by race and ethnicity. Among patients who arrived within 4.5 hours, Black and Hispanic patients had significantly lower thrombolysis treatment rates (pre-TS: AORs, 0.74 [95% CI, 0.70-0.79] for Black and 0.83 [95% CI, 0.76-0.90] for Hispanic; PS:III: AORs, 0.76 [95% CI, 0.74-0.78] for Black and 0.86 [95% CI, 0.83-0.89] for Hispanic). Compared with White patients, Black patients had slower treatment speed in both periods (pre-TS DTN≤60 minutes: AOR, 0.78 [95% CI, 0.71-0.87]; TS:III DTN≤60 minutes: AOR, 0.78 [95% CI, 0.75-0.81]), while Hispanic patients had significantly slower treatment speed during TS:III (AOR, 0.87; 95% CI, 0.83-0.92) but not pre-TS (AOR, 0.89; 95% CI, 0.77-1.02). For Asian patients, wide CIs precluded reliable analysis of the pre-TS period, but they showed lower odds of receiving IVT treatment in TS:III than White patients. Additionally, in TS:III, relative to White patients, odds of thrombolysis treatment with DTN times of 60, 45, and 30 minutes or less were more greatly reduced from pre-TS for Black patients than for Asian and Hispanic patients (Table 3).

With regard to clinical efficacy and safety outcomes, in TS:III, relative to White patients, the odds of discharge home were higher among Asian and Hispanic patients but lower among Black patients. The relative odds of independent ambulation at discharge were reduced among Black patients. In TS:III, in-hospital mortality was similar among Asian and Hispanic patients but lower among Black patients; in contrast, odds of combined mortality and hospice discharge were lower among Asian, Black, and Hispanic patients.

## Discussion

This national cohort study is the first to our knowledge to evaluate the association between 2-decade trends of thrombolysis disparities in delayed arrival and thrombolytic treatment for eligible patients by race and ethnicity through the ongoing TS national quality initiative. The results show that TS was associated with large and continuous improvements in thrombolysis frequency, timeliness, and outcomes among patients arriving at GWTG-Stroke hospitals within 4.5 hours. Disparities were not evident in thrombolysis metrics in unadjusted analysis but emerged after adjusting for patient and hospital characteristics, with Asian, Black, and Hispanic patients being less likely to be treated with thrombolysis or at faster DTN times than White patients. Furthermore, Asian, Black, and Hispanic patients were more likely to present after 4.5 hours, which is the most common exclusion criterion for thrombolysis.^34^

Target: Stroke was designed to facilitate hospital delivery of timely thrombolytic treatment.^8,9^ Metrics of success are the percentages of eligible patients treated within specified DTN times, and the goals have been advanced in each phase to achieve continuous improvement.^11,12,14^ Hospitals are provided with best practice strategies and recognized for meeting the national goals.^11,12,35^ The recognitions are based on rates for the entire hospital population without adjustment or specific targets for race, ethnicity, or health equity components. Over the 7-year period before TS, only one-quarter of patients treated with thrombolysis had DTN times of 60 minutes or less, and little improvement was observed.^31^ The initiation of TS was associated with a prompt and accelerated rate of increase of DTN times of 60 minutes or less in both TS:I and TS:II,^10,11,13^ with our study showing further increases in TS:III. Moreover, we observed that over the entire 2010-2021 TS period, thrombolysis rates, timeliness, and outcomes substantially and monotonically increased for all racial and ethnic groups. It is notable that the rate of improvement slowed during TS:III, which may be partially attributable to the concurrent COVID-19 pandemic. Although a prior study showed that DTN times in GWTG-Stroke hospitals did not change during the first few months of the pandemic,^36^ it might have limited the prioritization and resources for quality improvement.

This study identified important actionable disparities in stroke thrombolysis delivery nationwide. Time of hospital arrival is an important opportunity for the stroke system of care and health equity intervention. The past 2 decades have seen consistent racial and ethnic differences in hospital arrival times, with Black individuals and, to a lesser degree, Asian and Hispanic individuals disproportionately presenting after 4.5 hours, which excludes them from thrombolysis. Presentation after 4.5 hours may have been a major contributor of previously reported racial and ethnic disparities in thrombolysis rates but was not considered in prior studies due to the lack of data on arrival time.^15,16,17,18,19,20,21^ Studies using local data found that Black individuals were less likely than White individuals to present within 3 hours of stroke onset.^18,37^ A common reason for delay is extended time to activate EMS by patients and witnesses due to not recognizing the symptoms as serious, hesitation to call an ambulance for financial reasons, or taking a watch-and-wait approach.^38,39^ Targeted education initiatives for both the general population and by race and ethnicity to improve public and patient knowledge of stroke warning signs and the need to promptly activate EMS have occurred continuously over the past 25 years in the US and other countries, with mixed success.^40,41,42,43^ Based on experience with commercial advertising, it is critical for these campaigns to be constantly underway or else public knowledge may rapidly decline.^44^ Efforts to better understand and target barriers of message uptake and behavioral actions are highly desirable, such as the recently identified social environment paradox in which patients whose stroke onset is in the presence of nonrelatives arrive early while those with onset in the presence of spouses or family members arrive late.^45^ Further research and interventions in the patient population at risk may have profound outcomes for population health and health equity.

This study also found disparities in the speed of thrombolysis administration after arrival. Door-to-needle times within 60, 45, and 30 minutes improved substantially among all racial and ethnic groups, but the median DTN times remained longer in Asian, Hispanic, and Black individuals. Of note, there were no disparities in the arrival by 2 hours and treat by 3 hours or arrival by 3.5 hours and treat by 4.5 hours metrics. This pattern may have several sources, including slower deliberation among individuals of racial and ethnic minority groups due to suspicion of the medical system, a desire to consult with more family members, and concern about costs, but with similar final decisions when forced to choose by closing time windows. Further research into the sources and amelioration of these postarrival delays is urgently needed.

This study reflects the complexity of using performance measures to identify health disparities. Unlike outcome comparison in which risk adjustment is needed,^46^ unadjusted data are often used for recognition or health equity reporting of performance measures in part because they are more readily generated to inform continuous quality improvement.^47^ The findings of our study suggest caution in using only unadjusted data. Disparities in key thrombolysis metrics were not evident in unadjusted data but were recognized in adjusted analysis for Asian, Black, and Hispanic patients. Consideration should be given to report risk-adjusted hospital performance by race and ethnicity. Furthermore, specific interventions are needed to reduce these disparities while improving care for all.

### Limitations

This study has several limitations. First, participation in GWTG-Stroke is voluntary, and data are self-reported by participating hospitals. However, prior quality audits of GWTG-Stroke data have shown high concordance rates with source documentation.^25^ Second, Asian and Hispanic classifications could not be further disaggregated, which may mask potential differences within these groups. Third, small and rural hospitals are underrepresented in GWTG-Stroke, even though their participation has been increasing over time. Fourth, changes over time may be influenced by the changing participation of hospitals within the study. Fifth, it is possible that the improved thrombolysis metrics have been influenced by factors other than TS. However, efforts in place during the 2003-2009 period were observed to have little influence on DTN times.^8,11^ Sixth, patients with missing LKW data were excluded from the analyses, which may limit conclusions drawn from the results. Seventh, because the study was retrospective and observational, we cannot definitively show a causal relationship between TS and improved outcomes as secular trends at TS nonparticipating hospitals were not analyzed.

## Conclusions

The findings from this cohort study show that TS was associated with continuous improvement from 2009 to 2021 in thrombolysis frequency, timeliness, and functional outcomes for patients from all racial and ethnic groups presenting to GWTG-Stroke–participating hospitals within 4.5 hours. Racial and ethnic disparities were not evident in unadjusted quality metrics, but after adjustment for patient and hospital characteristics, Asian, Black, and Hispanic patients had lower odds of receiving thrombolysis and longer DTN times than White patients. Furthermore, Asian, Black, and Hispanic individuals were found to have higher odds of arriving at the hospital after the 4.5-hour thrombolysis time window compared with White individuals. Further improvement of the stroke system of care and health equity should focus on continued and improved prehospital community stroke education and readiness, understanding and resolution of sources of slower postarrival decision making, and incorporation of risk-adjusted quality measure reporting by race and ethnicity.

## Notes

Supplement 1. **eMethods.** Get With The Guidelines (GWTG)–Stroke Database, Target: Stroke, Statistical Analyses, and Missing Data **eFigure 1.** Target: Stroke Timelines, Goals, and Strategies **eFigure 2.** Study Flow Chart **eFigure 3.** Proportion of Patients Arriving After 4.5 Hours by Race and Ethnicity **eFigure 4.** Trends in Stroke Volume, Hospital Bed Size, and Number of Hospitals Participating to GWTG-Stroke **eFigure 5.** Trends in Thrombolysis Rates Among Patients Arriving Within 4.5 Hours by Race and Ethnicity **eFigure 6.** Trends in Thrombolysis Time Metrics by Race and Ethnicity **eFigure 7.** Trends of In-Hospital Outcomes Among Patients Receiving Intravenous Thrombolysis **eTable 1.** Unknown or Missing Last Known Well Time by Race and Ethnicity **eTable 2.** Missing Rates of Key Clinical Characteristics by Race and Ethnicity **eTable 3.** Missing Rates of Key Clinical Characteristics by Race and Ethnicity Among Patients With NIHSS Recorded **eTable 4.** Descriptive Thrombolysis Metrics and Outcomes by Race and Ethnicity Over Time
eReferences

Supplement 2.
Data Sharing Statement
