# Supplementary material for: Trends in Stroke Thrombolysis Care Metrics and Outcomes by Race and Ethnicity, 2003-2021
Source: JAMA Netw Open. 2024 Feb 7;7(2):e2352927. doi: 10.1001/jamanetworkopen.2023.52927 (PMC10851100; doi:10.1001/jamanetworkopen.2023.52927)
Supplement: Supplement 1. — eMethods. Get With The Guidelines (GWTG)–Stroke Database, Target: Stroke, Statistical Analyses, and Missing Data eFigure 1. Target: Stroke Timelines, Goals, and Strategies eFigure 2. Study Flow Chart eFigure 3. Proportion of Patients Arriving After 4.5 Hours by Race and Ethnicity eFigure 4. Trends in Stroke Volume, Hospital Bed Size, and Number of Hospitals Participating to GWTG-Stroke eFigure 5. Trends in Thrombolysis Rates Among Patients Arriving Within 4.5 Hours by Race and Ethnicity eFigure 6. Trends in Thrombolysis Time Metrics by Race and Ethnicity eFigure 7. Trends of In-Hospital Outcomes Among Patients Receiving Intravenous Thrombolysis eTable 1. Unknown or Missing Last Known Well Time by Race and Ethnicity eTable 2. Missing Rates of Key Clinical Characteristics by Race and Ethnicity eTable 3. Missing Rates of Key Clinical Characteristics by Race and Ethnicity Among Patients With NIHSS Recorded eTable 4. Descriptive Thrombolysis Metrics and Outcomes by Race and Ethnicity Over Time eReferences [file jamanetwopen-e2352927-s001.pdf]

Supplemental Online Content

Man S, Solomon N, Mac Grory B, et al. Trends in stroke thrombolysis care metrics and outcomes by race and ethnicity, 2003-2021. *JAMA Netw Open*. 2024;7(1):e2352927. doi:10.1001/jamanetworkopen.2023.52927

eMethods. Get With The Guidelines (GWTG)–Stroke Database, Target: Stroke, Statistical Analyses, and Missing Data

eFigure 1. Target: Stroke Timelines, Goals, and Strategies

eFigure 2. Study Flow Chart

eFigure 3. Proportion of Patients Arriving After 4.5 Hours by Race and Ethnicity

eFigure 4. Trends in Stroke Volume, Hospital Bed Size, and Number of Hospitals Participating to GWTG-Stroke

eFigure 5. Trends in Thrombolysis Rates Among Patients Arriving Within 4.5 Hours by Race and Ethnicity

eFigure 6. Trends in Thrombolysis Time Metrics by Race and Ethnicity

eFigure 7. Trends of in-Hospital Outcomes Among Patients Receiving Intravenous Thrombolysis

eTable 1. Unknown or Missing Last Known Well Time by Race and Ethnicity

eTable 2. Missing Rates of Key Clinical Characteristics by Race and Ethnicity

eTable 3. Missing Rates of Key Clinical Characteristics by Race and Ethnicity Among Patients With NIHSS Recorded

eTable 4. Descriptive Thrombolysis Metrics and Outcomes by Race and Ethnicity Over Time

eReferences

This supplemental material has been provided by the authors to give readers additional information about their work.

© 2024 Man S et al. *JAMA Network Open.*

eMethods. Get With The Guidelines (GWTG)–Stroke Database, Target: Stroke, Statistical Analyses, and Missing Data

Get With The Guidelines (GWTG)-Stroke

GWTG-Stroke is an ongoing prospective data collection launched by the American Heart Association and American Stroke Association (AHA/ASA) to support continuous quality improvement of hospital systems of care. [1,2] Trained hospital personnel are instructed to collect clinical data including demographics, medical history, index stroke data, and in-hospital outcomes, of consecutive patients treated for acute ischemic stroke or TIA by either prospective clinical identification, retrospective identification using ICD-9 and ICD-10 codes, or a combination. [1,2] As an effort to monitor race/ethnic disparities in stroke care, race/ethnicity data in GWTG-Stroke was recorded by hospital staff from various sources, including patient self-designation, administrative personnel during the registration process, or nursing intake forms.[3–5] The data entry tool supports a multi-select option that includes single-race, multi-race, and ethnic categories, and a separate data element for Hispanic ethnicity (yes versus no/not documented).[3] Previous audit has shown that the overall accuracy of GWTG-Stroke was above 90% for most variables and time-related performance measures had excellent ($\kappa \geq 0 \cdot 75$) reliability.[2]

Target: Stroke

The initial Target: Stroke (Phase I) was launched in January 2010 by the AHA/ASA to improve acute stroke care with an initial focus on DTN times for IVT for eligible patients with acute ischemic stroke. The primary goal of Target: Stroke Phase I was for participating hospitals to achieve DTN within 60 minutes in at least 50% of their patients.[6] To achieve these goals, Target: Stroke provided participating hospitals with best practice strategies, supporting tools, and educational resources necessary to improve the timeliness of intravenous thrombolytic therapy to eligible patients. An expert working group performed a systematic review of the published data on improving DTN times and identified 10 best practice strategies that could be rapidly, feasibly, and cost effectively adopted by participating hospitals.[6,7] Target: Stroke phase II was launched in January 2014 to further improve DTN times with more aggressive targets and additional strategies.[8,9] The primary goal of Target: Stroke phase II was for hospitals to achieve DTN within 60 minutes in at least 75% of patients treated with intravenous thrombolysis and DTN within 45 minutes in at least 50% of patients. Target: Stroke Phase III was launched in January 2019 which set an even more aggressive targets for timely IVT administration with additional targets for endovascular thrombectomy.[10] The primary goal of Target: Stroke Phase III was to achieve DTN within 60 minutes in at least 85% patients treated with IVT. The secondary goal of Phase III was to achieve DTN within 45 minutes in 75% or more, and DTN within 30 minutes in 50% or more of patients treated with IVT. The recognitions were based on unadjusted gross rates for the entire hospital population with four levels: Target: Stroke Honor Roll (DTN times within 60 minutes for at least 75 percent of applicable patients), Target: Stroke Honor Roll-Elite (DTN times within 60 minutes for at least 85 percent of applicable patients), Target: Stroke Honor Roll-Elite Plus (DTN times within 45 minutes for at least 75 percent of applicable patients and DTN times within 30 minutes for at least 50 percent of applicable patients), and Target: Stroke Honor Roll Advanced Therapy (Door-to-device times in at least 50% of applicable patients within 90 minutes for direct arriving and within 60 minutes for transfers).[10]

Statistical Analyses

The analyses of thrombolytic treatment metrics and outcomes were conducted among patients arriving within 4.5 hours from stroke onset. The annual thrombolytic treatment rates and key metrics from 2003 to 2021 in Asian, Hispanics, Non-Hispanic Black (Black), and Non-Hispanic White (White) patients were depicted graphically. Logistic regression models were constructed to examine the gaps of thrombolysis rates and key metrics among Asian, Black, and Hispanic patients relative to White patients. We used stepwise adjustment approach, with Model 1, unadjusted; Model 2, adjusted for demographics, Model 3, added medical history; Model 4, added admission variables; and Model 5, added hospital factors (fully adjusted model). The adjusted odds of annual thrombolysis utilization and key metrics of each race and ethnicity were plotted. The models accounted for within and across-hospital variability with hospital-specific random intercepts. To account for nonlinear relationships between the continuous variables and the log odds of the outcomes, restricted cubic splines with four knots were applied to the continuous variables as appropriate.

Multivariable logistic regression models with random intercepts was constructed to compute a the odds of each outcome in TS:I (January 1, 2010-December 31, 2013), TS:II (January 1, 2014-December 31, 2018), and TS:III (January 1, 2019-December 31, 2021) versus the pre-TS period (January 1, 2003-December 31, 2009) within each

race and ethnicity group. Multivariable logistic regression model was constructed and the odds ratio was used to estimate the changes of the gaps between Asian, Black, and Hispanic versus White during the pre-TS period and the current phase (TS:III). The models were adjusted for the covariates as listed in the main text and restricted cubic splines were applied to continuous variables as needed. These patient and hospital variables have been used in prior GWTG-Stroke analyses and were demonstrated to be associated with outcomes.[11–16] Hospital-specific random intercepts were used to account for across and within-hospital clustering.

Missing data: Patients with missing data for sex, age, race/ethnicity, and thrombolysis timeliness were excluded from the study. Missing medical history values were imputed to "No". Hospital size was imputed to 430 for academic sites while non-academic sites was imputed to 210 based on the approximate teaching-specific median bed size in the registry. Patients from sites missing other hospital characteristics were excluded from the adjusted analyses. Covariates with ≥25% missingness were excluded in the study. Due to the size of this study, multiple imputation was computationally infeasible. Therefore, single imputation to the race-specific mode or median was conducted for remaining covariates with missing data. NIHSS was not imputed as it may not be missing at random. The adjusted analyses were conducted among patients with NIHSS documented. Sensitivity analyses were conducted without adjusting for NIHSS so that patients with missing NIHSS were included. The missing rates of key patient and hospital characteristics were low (eTable 2). The missing rates of key clinical characteristics among patients with NIHSS documented are provided in eTable 3.
All statistical analyses were performed using SAS Version 9.4 software (SAS Institute). All tests were 2-sided, with $P<0.05$ considered statistically significant.

© 2024 Man S et al. *JAMA Network Open.*

eFigure 1. Target: Stroke Timelines, Goals, and Strategies

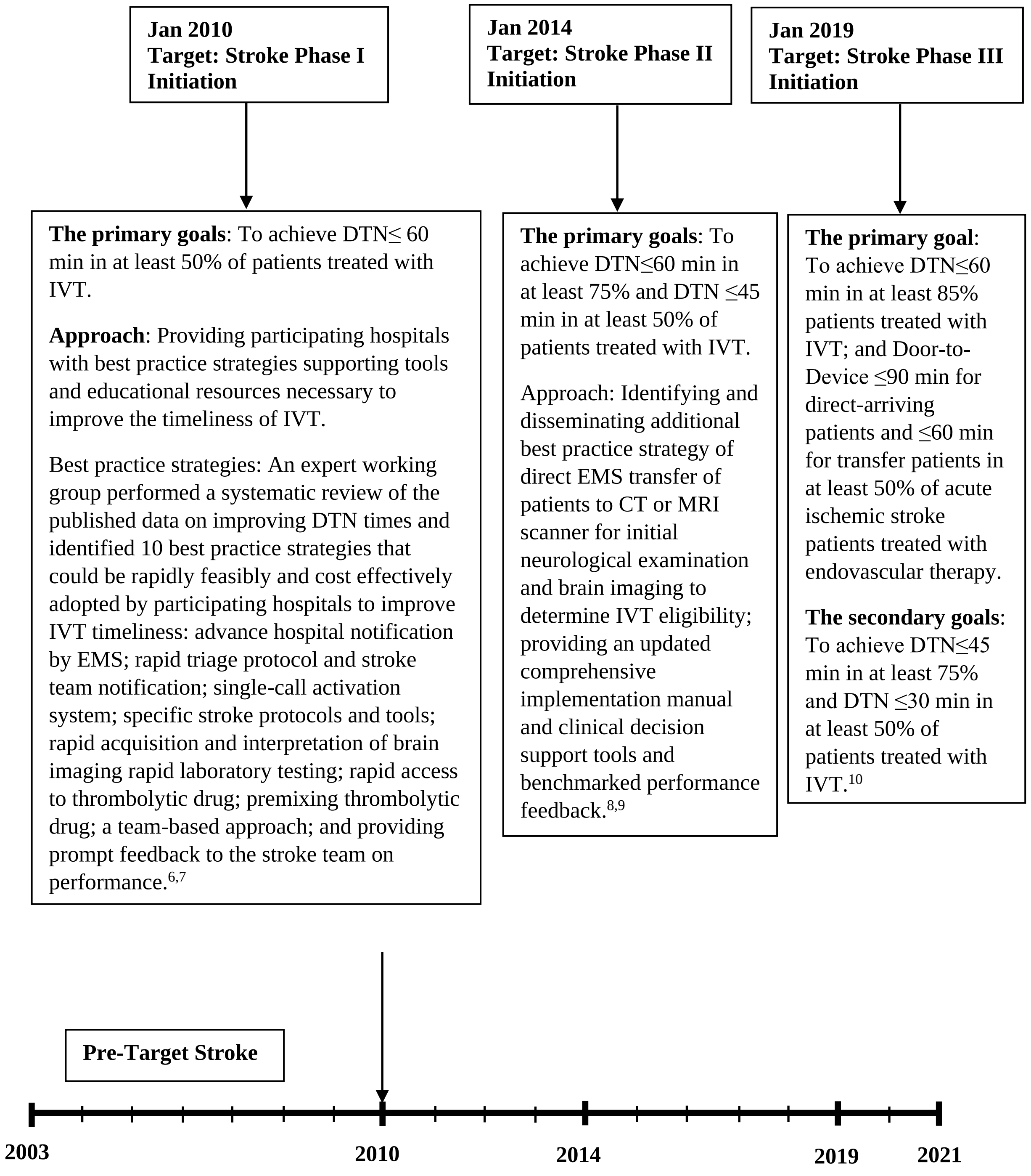

Abbreviations: DTN, door-to-needle; IVT, intravenous thrombolysis; EMS, emergency medical services; CT, computed tomographic; MRI, magnetic resonance imaging.

© 2024 Man S et al. *JAMA Network Open.*

eFigure 2. Study Flow Chart.

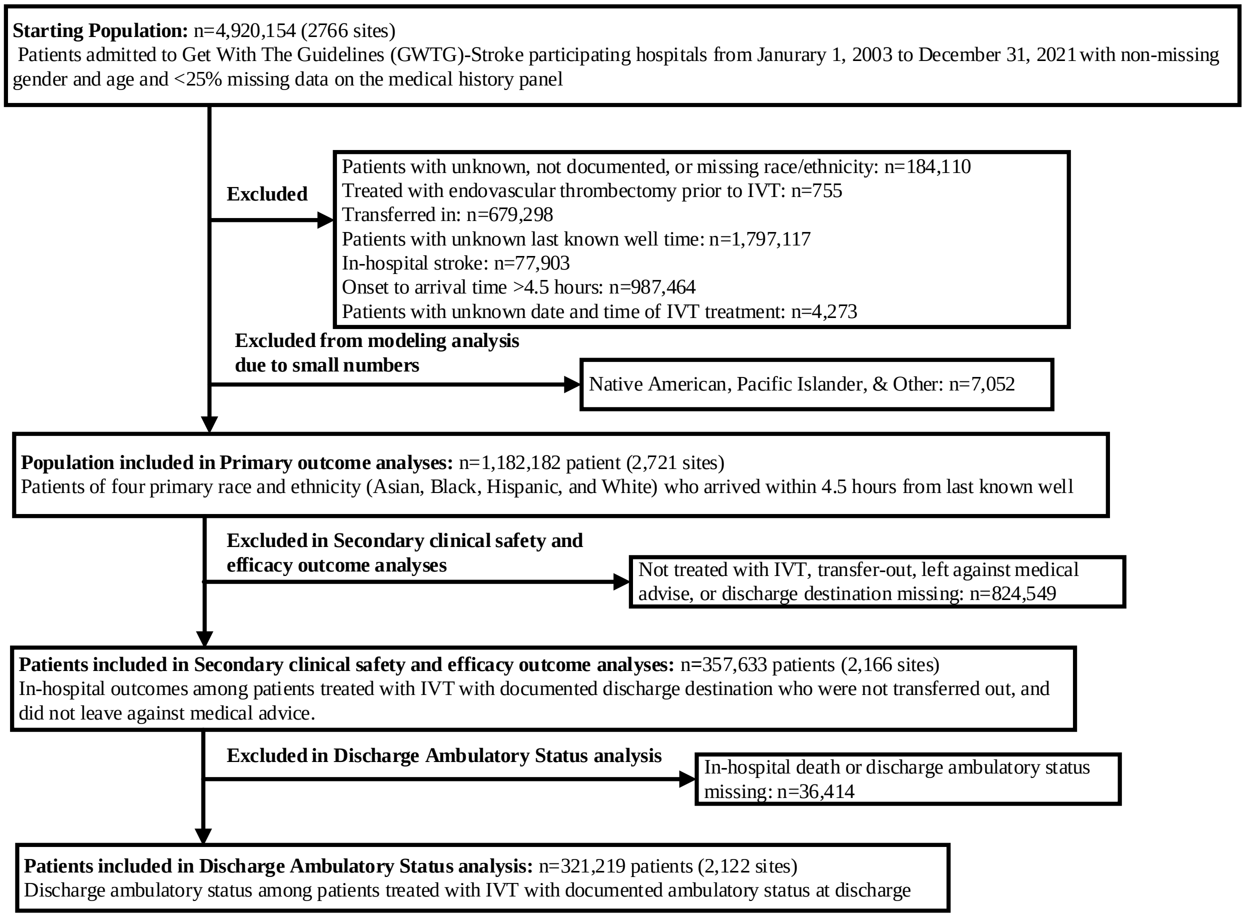

Patients included in the analysis stratified by Onset-to-Arrival time[b]

OTA time	Overall (N=2,148,074)	Asian (N=67,208)	Black (N=362,584)	Hispanic (N=165,303)	NA/PI/Other[a] (N=13,277)	White (N=1,539,702)
>4.5hrs	958840 (44.6%)	33833 (50.3%)	182269 (50.3%)	78472 (47.5%)	6225 (46.9%)	658041 (42.7%)
≤4.5hrs	1189234 (55.4%)	33375 (49.7%)	180315 (49.7%)	86831 (52.5%)	7052 (53.1%)	881661 (57.3%)

Legend

Abbreviations: IVT, intravenous thrombolysis; Black, non-Hispanic Black; NA, Native American; PI, Pacific Islander; White, Non-Hispanic White; OTA, Onset-to-Arrival.

[a] Other race and ethnicity included Native Hawaiian and those for whom "Specify Other Race" was selected on the Get With The Guideline-Stroke case report form.

[b]The study entry criteria were applied to the Onset-to-Arrival>4.5hr analysis which excluded patients with unknown or missing race/ethnicity, treated with endovascular therapy prior to IVT, transferred in, with unknown last known well time, in-hospital stroke, or unknown IVT treatment date or time.

© 2024 Man S et al. *JAMA Network Open.*

eFigure 3. Proportion of Patients Arriving After 4.5 Hours by Race and Ethnicity

A. Rates of Onset-to-Arrival>4.5 hours among all ischemic stroke patients

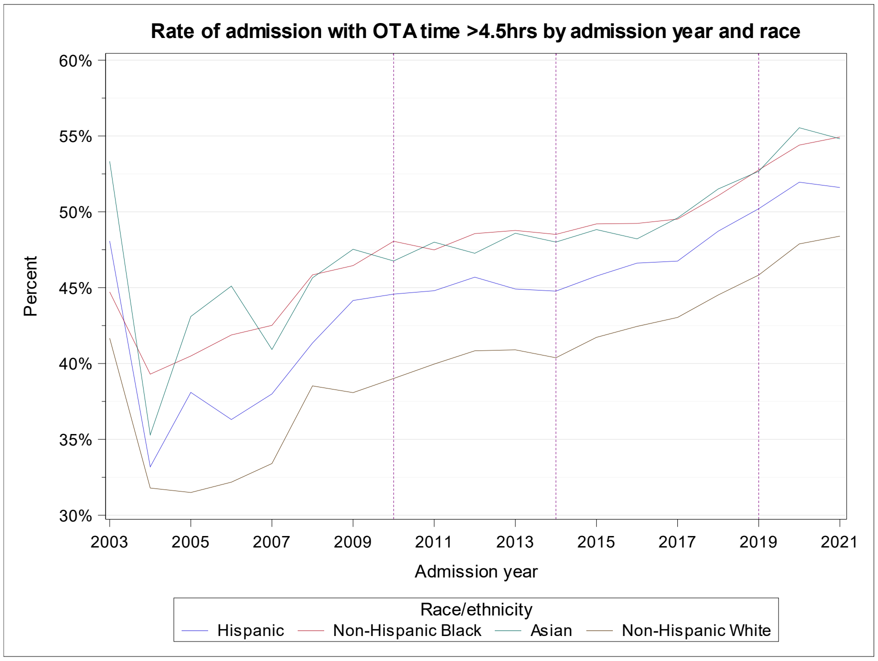

B. Rates of Onset-to-Arrival>4.5 hours among patients with moderate to severe stroke (NIHSS ≥5)

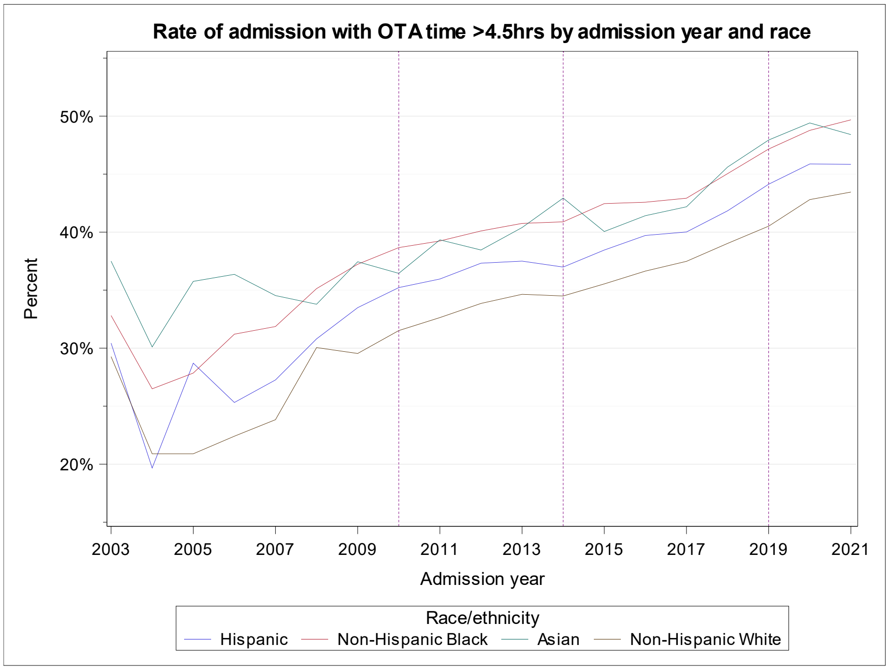

Abbreviations: OTA, onset-to-arrival; NIHSS, National Institutes of Health Stroke Scale.

© 2024 Man S et al. *JAMA Network Open.*

eFigure 4. Trends in Stroke Volume, Hospital Bed Size, and Number of Hospitals Participating to GWTG-Stroke

A. Number of hospitals participating to GWTG-Stroke

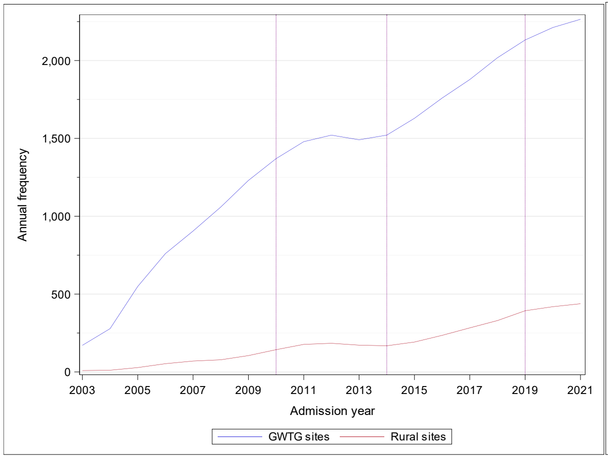

B. Trend of bedsize of hospitals participating to GWTG-Stroke

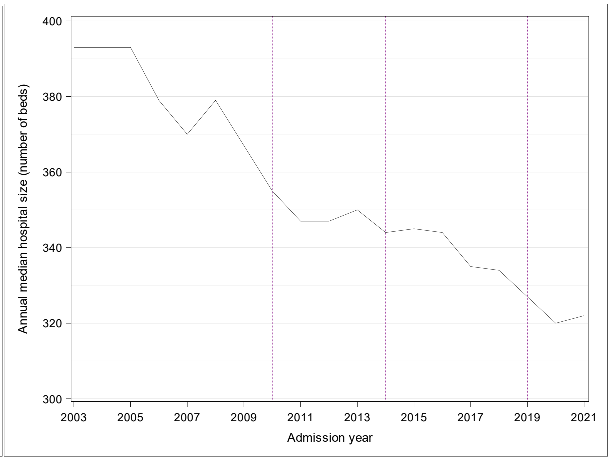

C. Annual ischemic stroke volume treated in GWTG-Stroke hospitals

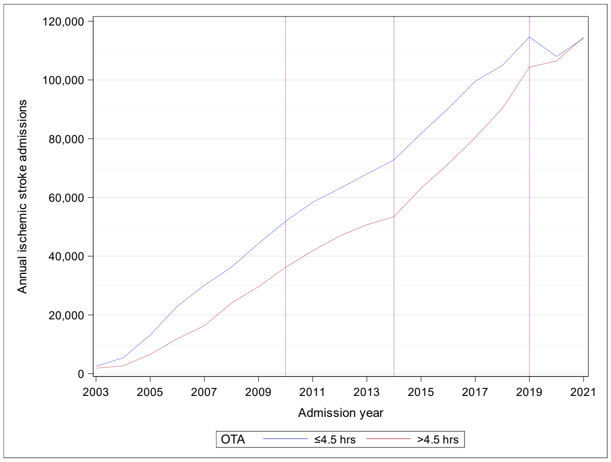

Abbreviations: GWTG, Get With The Guidelines.

© 2024 Man S et al. *JAMA Network Open.*

eFigure 5. Trends in Thrombolysis Rates Among Patients Arriving Within 4.5 Hours by Race and Ethnicity

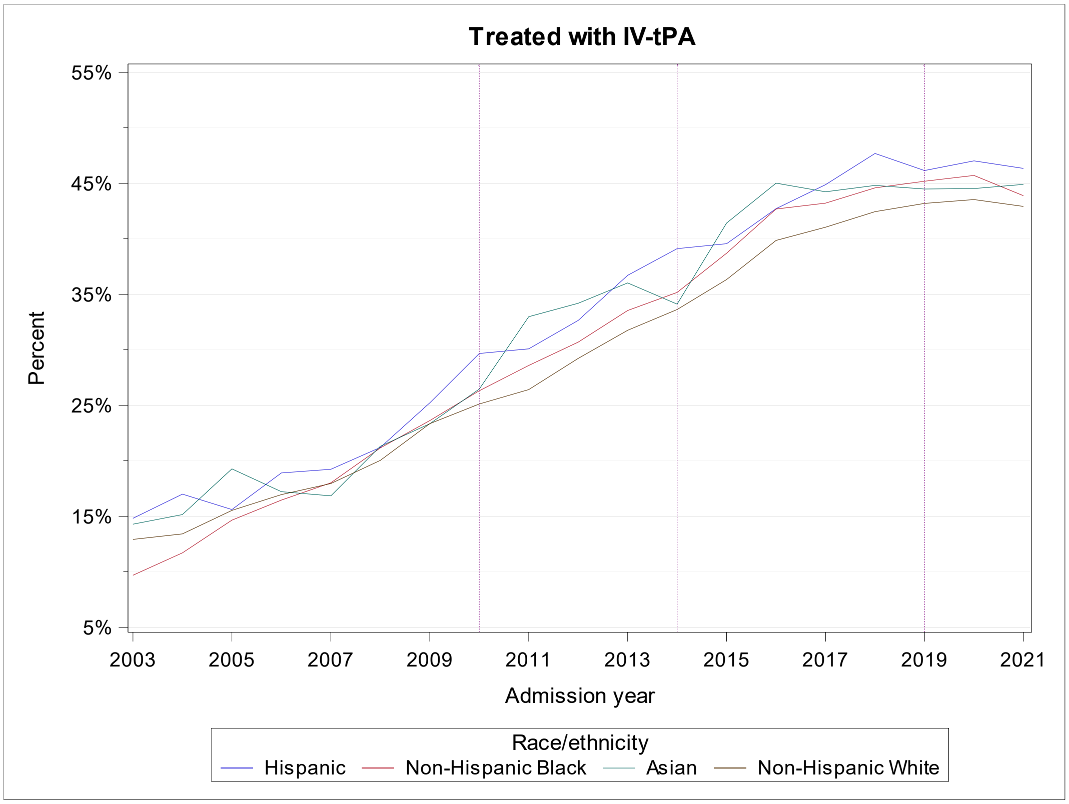

© 2024 Man S et al. *JAMA Network Open.*

eFigure 6. Trends in Thrombolysis Time Metrics by Race and Ethnicity

A. Proportion of DTN≤30, 45, and 60 minutes

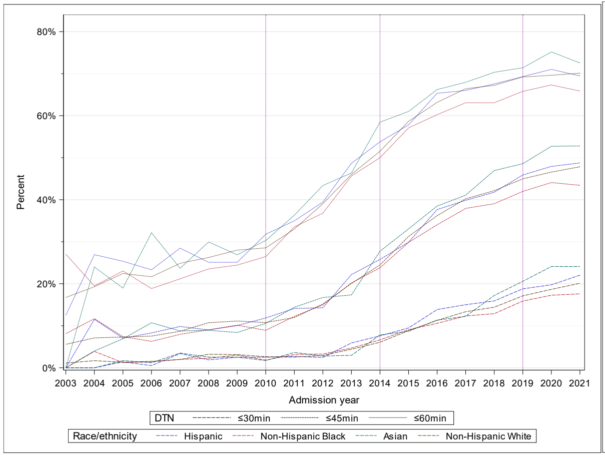

B. Arrival by 2 hours/Treat by 3 hours

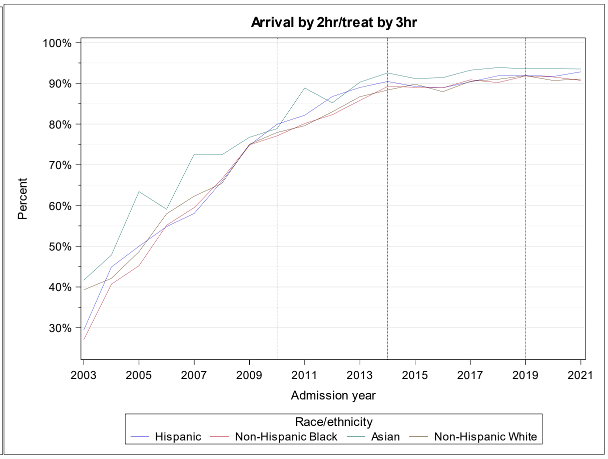

C. Arrival by 3.5 hours/Treat by 4.5 hours

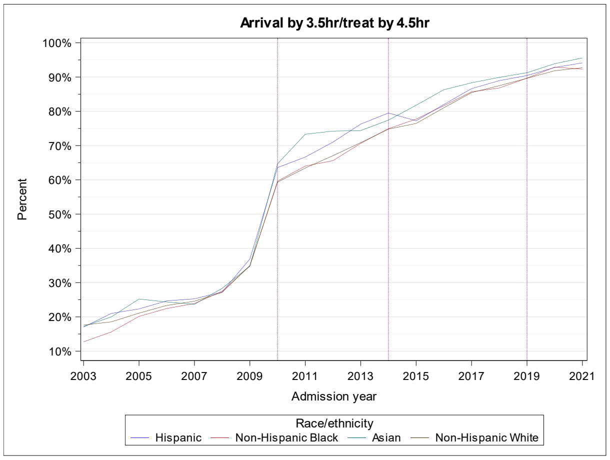

Abbreviations: DTN, Door-to-needle.

Legend

A. Proportion of thrombolytic administration with DTN≤30, 45, and 60 minutes among patients treated with intravenous thrombolysis within 4.5 hours; B. Proportion of patients treated with thrombolysis within 3 hours among those arriving within 2 hours without thrombolysis contraindications; C. Proportion of patients treated with thrombolysis within 4.5 hours among those arriving within 3.5 hours without thrombolysis contraindications.

© 2024 Man S et al. *JAMA Network Open.*

eFigure 7. Trends of In-Hospital Outcomes Among Patients Receiving Intravenous Thrombolysis

A. In-hospital mortality rate

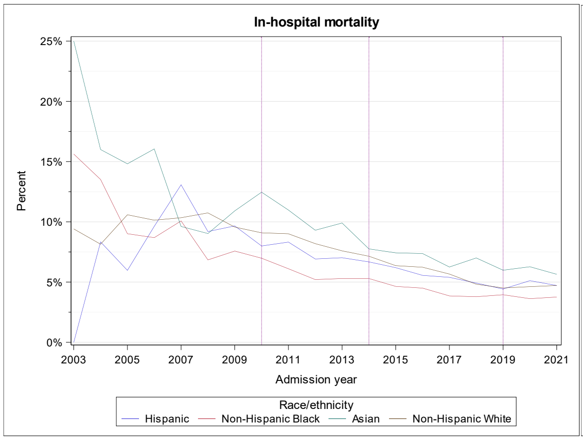

B. In-hospital mortality and discharge to hospice rate

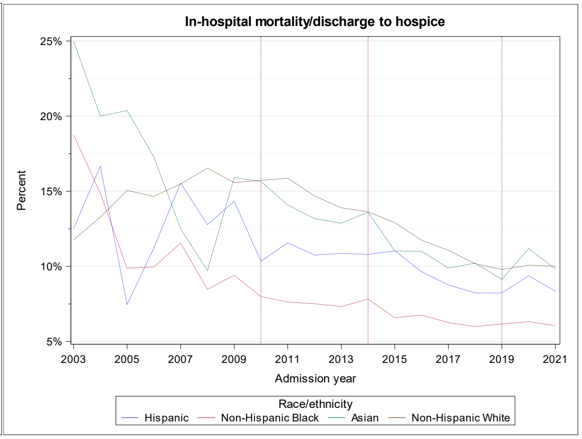

C. Discharged-to-home rate

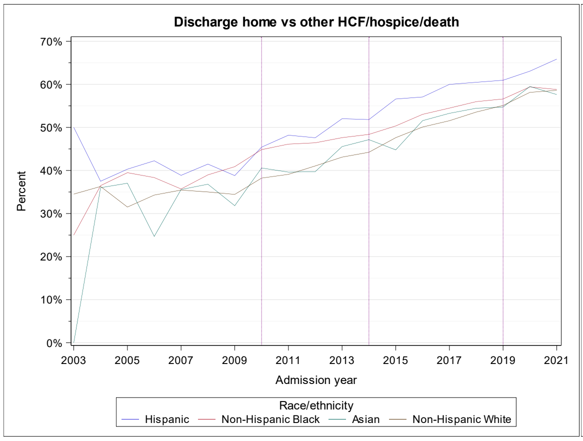

D. Ambulatory independently at discharge*

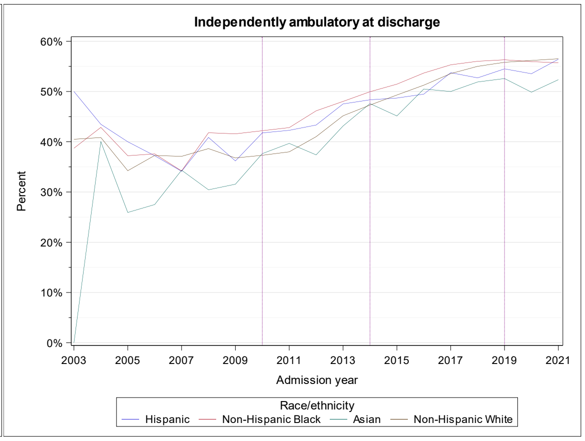

Legend

*Ambulatory independently at discharge were analyzed among patients who were discharged alive with ambulatory status documented).

© 2024 Man S et al. *JAMA Network Open.*

eTable 1. Unknown or Missing Last Known Well Time by Race and Ethnicity

	**Unknown LKW (N=1,010,757)**	**Missing LKW (N=786,360)**	**Documented LKW (N=2,258,874)**	**Std. Diff.[a] %**
Race/ethnicity				9.3
Asian	31,105 (3.1)	24,696 (3.1)	70,650 (3.1)	
Black	196,417 (19.4)	160,693 (20.4)	381,067 (16.9)	
Hispanic	70,973 (7.0)	59,548 (7.6)	173,815 (7.7)	
Native American, Pacific Islander, Other[b]	6,474 (0.6)	4,468 (0.6)	14,050 (0.6)	
White	705,788 (69.8)	536,955 (68.3)	1,619,292 (71.7)	

Abbreviation: LKW, last known well; Std. Diff., Standardized Difference.

[a]Standardized difference was calculated to measure the balance of racial-ethnic composition between the group of patients with Missing LKW time and those with documented LKW time. Standardized difference greater than 10% was considered significant imbalance.

[b]Other race and ethnicity included Native Hawaiian and those for whom "Specify Other Race" was selected on the Get With The Guideline-Stroke case report form.

Data are expressed as number of observations (%).

© 2024 Man S et al. *JAMA Network Open.*

eTable 2. Missing Rates of Key Clinical Characteristics by Race and Ethnicity

Factor	Overall (N=1182182)	Asian (N=33375)	Black (N=180315)	Hispanic (N=86831)	White (N=881661)
Age	0 (0.0)	0 (0.0)	0 (0.0)	0 (0.0)	0 (0.0)
Sex	0 (0.0)	0 (0.0)	0 (0.0)	0 (0.0)	0 (0.0)
Insurance	237991 (20.1)	7541 (22.6)	34735 (19.3)	19509 (22.5)	176206 (20.0)
Afib/flutter	7791 (0.7)	180 (0.5)	1119 (0.6)	864 (1.0)	5628 (0.6)
Prior Stroke/TIA	7791 (0.7)	180 (0.5)	1119 (0.6)	864 (1.0)	5628 (0.6)
CAD/Prior MI	7791 (0.7)	180 (0.5)	1119 (0.6)	864 (1.0)	5628 (0.6)
Carotid stenosis	7791 (0.7)	180 (0.5)	1119 (0.6)	864 (1.0)	5628 (0.6)
Diabetes mellitus	7791 (0.7)	180 (0.5)	1119 (0.6)	864 (1.0)	5628 (0.6)
Peripheral vessel disease	7791 (0.7)	180 (0.5)	1119 (0.6)	864 (1.0)	5628 (0.6)
Hypertension	7791 (0.7)	180 (0.5)	1119 (0.6)	864 (1.0)	5628 (0.6)
Dyslipidemia	7791 (0.7)	180 (0.5)	1119 (0.6)	864 (1.0)	5628 (0.6)
Heart failure	7791 (0.7)	180 (0.5)	1119 (0.6)	864 (1.0)	5628 (0.6)
Renal insufficiency	7791 (0.7)	180 (0.5)	1119 (0.6)	864 (1.0)	5628 (0.6)
Smoking	7791 (0.7)	180 (0.5)	1119 (0.6)	864 (1.0)	5628 (0.6)
Antiplatelet/anticoagulant before admission	53366 (4.5)	1560 (4.7)	7033 (3.9)	4253 (4.9)	40520 (4.6)
Arrival by EMS	36121 (3.1)	1060 (3.2)	5103 (2.8)	2388 (2.8)	27570 (3.1)
Off-hour arrival[a]	0 (0.0)	0 (0.0)	0 (0.0)	0 (0.0)	0 (0.0)
NIHSS	128643 (10.9)	3289 (9.9)	18379 (10.2)	7565 (8.7)	99410 (11.3)
Admission SBP	326100 (27.6)	9013 (27.0)	46129 (25.6)	21573 (24.8)	249385 (28.3)
Admission glucose	334753 (28.3)	9391 (28.1)	47074 (26.1)	22669 (26.1)	255619 (29.0)
Census region	0 (0.0)	0 (0.0)	0 (0.0)	0 (0.0)	0 (0.0)
Rural location	409 (0.0)	1 (0.0)	34 (0.0)	19 (0.0)	355 (0.0)
Teaching hospital	15274 (1.3)	921 (2.8)	2472 (1.4)	2075 (2.4)	9806 (1.1)
Number of hospital beds	17118 (1.4)	883 (2.6)	2688 (1.5)	2226 (2.6)	11321 (1.3)
Annual ischemic stroke volume	0 (0.0)	0 (0.0)	0 (0.0)	0 (0.0)	0 (0.0)
Annual IVT volume	0 (0.0)	0 (0.0)	0 (0.0)	0 (0.0)	0 (0.0)
Stroke center status	0 (0.0)	0 (0.0)	0 (0.0)	0 (0.0)	0 (0.0)

Abbreviations: Afib, atrial fibrillation; TIA, transient ischemic attack; CAD, coronary artery disease; MI, myocardial infarction; PVD, peripheral vessel disease; EMS, emergency medical service; NIHSS, National Institutes of Health Stroke Scale; SBP, systolic blood pressure; IVT, intravenous thrombolysis.

[a] Off hours: Regular working hours are defined as 7AM-6PM, Monday to Friday on non-holidays. Arriving outside of these hours are considered off hours.

Data are expressed as number of observations (%).

© 2024 Man S et al. *JAMA Network Open.*

eTable 3. Missing Rates of Key Clinical Characteristics by Race and Ethnicity Among Patients with NIHSS Recorded

Factor	Overall (N=1053539)	Asian (N=30086)	Black (N=161936)	Hispanic (N=79266)	White (N=782251)
Age	0 (0.0)	0 (0.0)	0 (0.0)	0 (0.0)	0 (0.0)
Sex	0 (0.0)	0 (0.0)	0 (0.0)	0 (0.0)	0 (0.0)
Insurance	194052 (18.4)	6370 (21.2)	28808 (17.8)	17126 (21.6)	141748 (18.1)
Afib/flutter	5062 (0.5)	126 (0.4)	727 (0.4)	623 (0.8)	3586 (0.5)
Prior Stroke/TIA	5062 (0.5)	126 (0.4)	727 (0.4)	623 (0.8)	3586 (0.5)
CAD/Prior MI	5062 (0.5)	126 (0.4)	727 (0.4)	623 (0.8)	3586 (0.5)
Carotid stenosis	5062 (0.5)	126 (0.4)	727 (0.4)	623 (0.8)	3586 (0.5)
Diabetes mellitus	5062 (0.5)	126 (0.4)	727 (0.4)	623 (0.8)	3586 (0.5)
Peripheral vessel disease	5062 (0.5)	126 (0.4)	727 (0.4)	623 (0.8)	3586 (0.5)
Hypertension	5062 (0.5)	126 (0.4)	727 (0.4)	623 (0.8)	3586 (0.5)
Dyslipidemia	5062 (0.5)	126 (0.4)	727 (0.4)	623 (0.8)	3586 (0.5)
Heart failure	5062 (0.5)	126 (0.4)	727 (0.4)	623 (0.8)	3586 (0.5)
Renal insufficiency	5062 (0.5)	126 (0.4)	727 (0.4)	623 (0.8)	3586 (0.5)
Smoking	5062 (0.5)	126 (0.4)	727 (0.4)	623 (0.8)	3586 (0.5)
Antiplatelet/anticoagulant before admission	44107 (4.2)	1285 (4.3)	5763 (3.6)	3608 (4.6)	33451 (4.3)
Arrival by EMS	27793 (2.6)	779 (2.6)	4017 (2.5)	1965 (2.5)	21032 (2.7)
Off-hour arrival[a]	0 (0.0)	0 (0.0)	0 (0.0)	0 (0.0)	0 (0.0)
NIHSS	0 (0.0)	0 (0.0)	0 (0.0)	0 (0.0)	0 (0.0)
Admission SBP	260992 (24.8)	7399 (24.6)	37267 (23.0)	17966 (22.7)	198360 (25.4)
Admission glucose	265971 (25.2)	7703 (25.6)	37734 (23.3)	18906 (23.9)	201628 (25.8)
Census region	0 (0.0)	0 (0.0)	0 (0.0)	0 (0.0)	0 (0.0)
Rural location	267 (0.0)	0 (0.0)	22 (0.0)	6 (0.0)	239 (0.0)
Teaching hospital	13177 (1.3)	782 (2.6)	2118 (1.3)	1829 (2.3)	8448 (1.1)
Number of hospital beds	15121 (1.4)	754 (2.5)	2334 (1.4)	2047 (2.6)	9986 (1.3)
Annual ischemic stroke volume	0 (0.0)	0 (0.0)	0 (0.0)	0 (0.0)	0 (0.0)
Annual IVT volume	0 (0.0)	0 (0.0)	0 (0.0)	0 (0.0)	0 (0.0)
Stroke center status	0 (0.0)	0 (0.0)	0 (0.0)	0 (0.0)	0 (0.0)

Abbreviations: Afib, atrial fibrillation; TIA, transient ischemic attack; CAD, coronary artery disease; MI, myocardial infarction; EMS, emergency medical service; NIHSS, National Institutes of Health Stroke Scale; SBP, systolic blood pressure; IVT, intravenous thrombolysis.

[a] Off hours: Regular working hours are defined as 7AM-6PM, Monday to Friday on non-holidays. Arriving outside of these hours are considered off hours.

Data are expressed as number of observations (%)

© 2024 Man S et al. *JAMA Network Open.*

eTable 4. Descriptive Thrombolysis Metrics and Outcomes by Race and Ethnicity Over Time

	2003-2009 Pre-TS	2010-2013 TS:I	2014-2018 TS:II	2019-2021 TS:III
**IVT treatment metrics**				
IVT Rate				
Asian	681/3423 (19.9)	2092/6371 (32.8)	5649/13311 (42.4)	4585/10270 (44.6)
Black	3988/20389 (19.6)	10502/34943 (30.1)	29245/70695 (41.4)	24379/54288 (44.9)
Hispanic	1748/8157 (21.4)	5518/16926 (32.6)	14895/34417 (43.3)	12705/27331 (46.5)
White	23744/122788 (19.3)	51919/183085 (28.4)	129189/330892 (39.0)	105804/244896 (43.2)
Door-to-Needle (DTN) times, min, median [Q1-Q3]				
Asian	77 [60-100]	67 [52-90]	51 [38-70]	45 [32-63]
Black	82 [63-105]	71 [53-95]	55 [40-76]	50 [36-69]
Hispanic	79 [60-102]	69 [52-93]	53 [39-72]	47 [33-66]
White	79 [60-100]	70 [53-92]	53 [39-72]	48 [34-66]
DTN ≤30min				
Asian	16/681 (2.3)	60/2092 (2.9)	685/5649 (12.1)	1052/4585 (22.9)
Black	93/3988 (2.3)	360/10502 (3.4)	3161/29245 (10.8)	4121/24379 (16.9)
Hispanic	38/1748 (2.2)	202/5518 (3.7)	1949/14895 (13.1)	2571/12705 (20.2)
White	605/23744 (2.5)	1687/51919 (3.2)	14722/129189 (11.4)	19704/105804 (18.6)
DTN ≤45min				
Asian	58/681 (8.5)	321/2092 (15.3)	2199/5649 (38.9)	2356/4585 (51.4)
Black	353/3988 (8.9)	1561/10502 (14.9)	9996/29245 (34.2)	10519/24379 (43.1)
Hispanic	164/1748 (9.4)	904/5518 (16.4)	5414/14895 (36.3)	6040/12705 (47.5)
White	2300/23744 (9.7)	7861/51919 (15.1)	46670/129189 (36.1)	49147/105804 (46.5)
DTN ≤60min				
Asian	182/681 (26.7)	847/2092 (40.5)	3715/5649 (65.8)	3346/4585 (73.0)
Black	909/3988 (22.8)	3877/10502 (36.9)	17475/29245 (59.8)	16167/24379 (66.3)
Hispanic	445/1748 (25.5)	2210/5518 (40.1)	9422/14895 (63.3)	8882/12705 (69.9)
White	6040/23744 (25.4)	19658/51919 (37.9)	80771/129189 (62.5)	73689/105804 (69.6)
DTN >60min				
Asian	499/681 (73.3)	1245/2092 (59.5)	1934/5649 (34.2)	1239/4585 (27.0)
Black	3079/3988 (77.2)	6625/10502 (63.1)	11770/29245 (40.2)	8212/24379 (33.7)
Hispanic	1303/1748 (74.5)	3308/5518 (59.9)	5473/14895 (36.7)	3823/12705 (30.1)
White	17704/23744 (74.6)	32261/51919 (62.1)	48418/129189 (37.5)	32115/105804 (30.4)
Arrival by 2 hours/Treat by 3 hours				
Asian	613/885 (69.3)	1642/1898 (86.5)	4115/4448 (92.5)	3095/3308 (93.6)
Black	3451/5522 (62.5)	8020/9806 (81.8)	21480/23935 (89.7)	16747/18329 (91.4)
Hispanic	1531/2377 (64.4)	4299/5056 (85.0)	11355/12579 (90.3)	8861/9612 (92.2)
White	21153/33321 (63.5)	40934/49757 (82.3)	97897/109216 (89.6)	74538/81712 (91.2)
Arrival by 3.5 hours/Treat by 4.5 hours				
Asian	673/2422 (27.8)	2014/2787 (72.3)	5331/6236 (85.5)	4238/4527 (93.6)
Black	3943/14702 (26.8)	10166/15529 (65.5)	27770/33820 (82.1)	22661/24746 (91.6)
Hispanic	1722/5934 (29.0)	5343/7626 (70.1)	14313/17136 (83.5)	11819/12782 (92.5)
White	23527/86910 (27.1)	50254/76527 (65.7)	123483/151146 (81.7)	99150/108519 (91.4)
Patient or family refusal of IVT				
Asian	108/3423 (3.2)	232/6371 (3.6)	424/13311 (3.2)	419/10270 (4.1)
Black	623/20389 (3.1)	1325/34943 (3.8)	2843/70695 (4.0)	2191/54288 (4.0)
Hispanic	259/8157 (3.2)	593/16926 (3.5)	1156/34417 (3.4)	994/27331 (3.6)

© 2024 Man S et al. *JAMA Network Open.*

	2003-2009 Pre-TS	2010-2013 TS:I	2014-2018 TS:II	2019-2021 TS:III
White	4626/122788 (3.8)	7912/183085 (4.3)	13951/330892 (4.2)	10315/244896 (4.2)
Arrival by Emergency Medical Service				
Asian	2424/3328 (72.8)	4009/5539 (72.4)	9278/13246 (70.0)	7077/10202 (69.4)
Black	14526/19627 (74.0)	23545/31567 (74.6)	50806/70138 (72.4)	38937/53880 (72.3)
Hispanic	5771/7872 (73.3)	10733/15209 (70.6)	22847/34223 (66.8)	17921/27139 (66.0)
White	89025/118544 (75.1)	121139/163386 (74.1)	236510/328804 (71.9)	171515/243357 (70.5)
**In-Hospital Outcomes among patients treated with IVT**				
Symptomatic intracranial hemorrhage within 36 hours				
Asian	50/681 (7.3)	117/2092 (5.6)	229/5649 (4.1)	146/4585 (3.2)
Black	235/3988 (5.9)	408/10502 (3.9)	900/29245 (3.1)	717/24379 (2.9)
Hispanic	99/1748 (5.7)	272/5518 (4.9)	464/14895 (3.1)	390/12705 (3.1)
White	1272/23744 (5.4)	2251/51919 (4.3)	4111/129189 (3.2)	3095/105804 (2.9)
Any thrombolytic complications				
Asian	68/681 (10.0)	206/2092 (9.8)	417/5649 (7.4)	264/4585 (5.8)
Black	341/3988 (8.6)	885/10502 (8.4)	1853/29245 (6.3)	1375/24379 (5.6)
Hispanic	150/1748 (8.6)	490/5518 (8.9)	902/14895 (6.1)	737/12705 (5.8)
White	1856/23744 (7.8)	4222/51919 (8.1)	7907/129189 (6.1)	5851/105804 (5.5)
In-hospital mortality rate				
Asian	73/632 (11.6)	193/1854 (10.4)	338/4771 (7.1)	231/3885 (5.9)
Black	311/3767 (8.3)	547/9472 (5.8)	1072/24915 (4.3)	755/19982 (3.8)
Hispanic	162/1636 (9.9)	374/5012 (7.5)	721/12831 (5.6)	514/10836 (4.7)
White	2225/22022 (10.1)	3819/45681 (8.4)	6342/107143 (5.9)	3839/83194 (4.6)
In-hospital mortality and discharge to hospice				
Asian	93/632 (14.7)	254/1854 (13.7)	520/4771 (10.9)	389/3885 (10.0)
Black	371/3767 (9.8)	717/9472 (7.6)	1636/24915 (6.6)	1235/19982 (6.2)
Hispanic	221/1636 (13.5)	546/5012 (10.9)	1216/12831 (9.5)	935/10836 (8.6)
White	3420/22022 (15.5)	6810/45681 (14.9)	12528/107143 (11.7)	8287/83194 (10.0)
Discharge-to-home rate				
Asian	209/632 (33.1)	774/1854 (41.7)	2427/4771 (50.9)	2224/3885 (57.2)
Black	1464/3767 (38.9)	4398/9472 (46.4)	13205/24915 (53.0)	11639/19982 (58.2)
Hispanic	655/1636 (40.0)	2444/5012 (48.8)	7414/12831 (57.8)	6865/10836 (63.4)
White	7616/22022 (34.6)	18594/45681 (40.7)	53524/107143 (50.0)	47630/83194 (57.3)
Independent ambulation at discharge among patients with discharge ambulatory status documented				
Asian	187/606 (30.9)	553/1387 (39.9)	2184/4420 (49.4)	1892/3662 (51.7)
Black	1391/3517 (39.6)	3397/7514 (45.2)	12347/22962 (53.8)	10302/18399 (56.0)
Hispanic	556/1481 (37.5)	1649/3738 (44.1)	5967/11688 (51.1)	5603/10208 (54.9)
White	7611/20393 (37.3)	14495/35460 (40.9)	51138/98712 (51.8)	43286/77072 (56.2)

Abbreviations: TS, Target: Stroke; IVT, intravenous thrombolysis; IQR, interquartile range; NIHSS, National Institutes of Health Stroke Scale; EMS, emergency medical service.

Data are expressed as number of observations (%), or median [Interquartile range]

© 2024 Man S et al. *JAMA Network Open.*

eReferences

1. Fonarow GC, Reeves MJ, Smith EE, et al. Characteristics, performance measures, and in-hospital outcomes of the first one million stroke and transient ischemic attack admissions in get with the guidelines-stroke. Circ Cardiovasc Qual Outcomes. 2010;3(3):291-302.
2. Xian Y, Fonarow GC, Reeves MJ, et al. Data quality in the American Heart Association Get With The Guidelines-Stroke (GWTG-Stroke): results from a national data validation audit. Am Heart J. 2012;163(3):392-8, 398.e1.
3. Schwamm LH, Reeves MJ, Pan W, et al. Race/Ethnicity, quality of care, and outcomes in ischemic stroke. Circulation. 2010;121(13):1492-1501.
4. Therrien M, Ramirez RR. The Hispanic Population in the United States Population Characteristics Helping You Make Informed Decisions: 2001. Accessed January 20, 2020. https://cps.ipums.org/cps/resources/cpr/p20-535.pdf
5. Mehta RH, Cox M, Smith EE, et al. Race/ethnic differences in the risk of hemorrhagic complications among patients with ischemic stroke receiving thrombolytic therapy. Stroke. 2014;45(8):2263-2269.
6. Fonarow GC, Pan W, Saver JL, et al. Comparison of 30-day mortality models for profiling hospital performance in acute ischemic stroke with vs without adjustment for stroke severity. JAMA. 2012;308(3):257-264.
7. Xian Y, Smith EE, Zhao X, et al. Strategies used by hospitals to improve speed of tissue-type plasminogen activator treatment in acute ischemic stroke. Stroke. 2014;45(5):1387-1395.
8. Xian Y, Xu H, Lytle B, et al. Use of Strategies to Improve Door-to-Needle Times With Tissue-Type Plasminogen Activator in Acute Ischemic Stroke in Clinical Practice: Findings from Target: Stroke. Circ Cardiovasc Qual Outcomes. 2017;10(1):e003227.
9. Fonarow GC, Zhao X, Smith EE, et al. Door-to-needle times for tissue plasminogen activator administration and clinical outcomes in acute ischemic stroke before and after a quality improvement initiative. JAMA. 2014;311(16):1632-1640.
10. American Heart Association. Introducing Target: Stroke Phase III. https://www.heart.org/en/professional/quality-improvement/target-stroke/introducing-target-stroke-phase-iii. Accessed February 5, 2023.
11. Fonarow GC, Pan W, Saver JL, et al. Comparison of 30-day mortality models for profiling hospital performance in acute ischemic stroke with vs without adjustment for stroke severity. JAMA. 2012;308(3):257-264.
12. Reeves MJ, Smith E, Fonarow G, et al. Off-hour admission and in-hospital stroke case fatality in the get with the guidelines-stroke program. Stroke. 2009;40(2):569-576.
13. Messe SR, Khatri P, Reeves MJ, et al. Why are acute ischemic stroke patients not receiving IV tPA? Results from a national registry. Neurology. 2016;87(15):1565-1574.
14. Fonarow GC, Smith EE, Saver JL, et al. Timeliness of tissue-type plasminogen activator therapy in acute ischemic stroke: Patient characteristics, hospital factors, and outcomes associated with door-to-needle times within 60 minutes. Circulation. 2011;123(7):750-758.
15. Smith EE, Shobha N, Dai D, et al. A risk score for in-hospital death in patients admitted with ischemic or hemorrhagic stroke. J Am Heart Assoc. 2013;2(1):e005207.
16. Smith EE, Shobha N, Dai D, et al. Risk score for in-hospital ischemic stroke mortality derived and validated within the Get With the Guidelines-Stroke Program. Circulation. 2010;122(15):1496-1504.

© 2024 Man S et al. *JAMA Network Open.*
