# Supplementary material for: Trends in Stroke Thrombolysis Care Metrics and Outcomes by Race and Ethnicity, 2003-2021
Source: JAMA Netw Open. 2024 Feb 7;7(2):e2352927. doi: 10.1001/jamanetworkopen.2023.52927 (PMC10851100; doi:10.1001/jamanetworkopen.2023.52927)
Supplement: Supplement 2. — Data Sharing Statement [file jamanetwopen-e2352927-s002.pdf]

Data Sharing Statement

Man. Trends in Stroke Thrombolysis Care Metrics and Outcomes by Race and Ethnicity, 2003-2021. *JAMA Netw Open*. Published February 07, 2024.
doi:10.1001/jamanetworkopen.2023.52927

Data
Data available: No

Additional Information
Explanation for why data not available: Additional Information Explanation for why data not available: Given that data were collected for clinical care and quality improvement, rather than primarily for research, data sharing agreements require an application process in order for other researchers to access the data. Researchers interested in utilizing the American Heart Association (AHA) Get With The Guidelines for research purposes, including for validation, can submit proposals at https://www.heart.org/en/professional/quality-improvement/quality-research-and-publications/national-level-program-data-research-opportunities. Additional information regarding the statistical analysis plan and analytic code may also be available from Duke Clinical Research Institute on request.
